# Robust Self‐Healing Metallo‐Supergels of Folic Acid: Potential Sustainable Gelator for Oilfield Applications

**DOI:** 10.1002/chem.202500748

**Published:** 2025-04-09

**Authors:** Mahya Asgharian Marzabad, Subhasis Chattopadhyay, Sami Hietala, Nonappa Nonappa, Radek Marek, Ondřej Jurček

**Affiliations:** ^1^ Department of Chemistry Faculty of Science Masaryk University Brno Czechia; ^2^ Department of Natural Drugs Faculty of Pharmacy Masaryk University Brno Czechia; ^3^ CEITEC – Central European Institute of Technology Masaryk University Brno Czechia; ^4^ Department of Chemistry University of Helsinki Helsinki Finland; ^5^ Faculty of Engineering and Natural Sciences Tampere University Tampere Finland

**Keywords:** folic acid, gel, metallogel, oil mining, rheology, supramolecular chemistry

## Abstract

The majority of known metallosupramolecular gels are based on carefully designed ligands using extensive chemical synthesis. Their gelation is often limited to a certain specific metal salt. We demonstrate that in the presence of a wide group of metal salts natural and readily available folic acid (FA) can act as a supergelator. We report a systematic investigation of 17 mechanically robust FA‐based metallogels at extremely low concentrations (<0.2 wt%). Using oscillatory rheological measurements, we further show that these metallogels undergo rapid recovery and self‐healing, recovering up to 95% of their original stiffness within 1 min. Among the metallogels studied, FA‐chromium(III) acetate gel (0.4 wt%) displayed the highest stiffness with a storage modulus of 4 kPa. More importantly, the stiffness, recovery, and sol ↔ gel transitions can be readily tuned by changing either the metal salt or the concentration. Using a combination of various analytical methods, we also suggest a structure of self‐assembly in the metallogel network. This study defines non‐toxic FA as a robust and sustainable building block for metallogels—mechanically tunable, multi‐responsive soft materials. Finally, as a proof‐of‐concept experiment, we demonstrate that the FA‐chromium(III) acetate gel can be considered as a potent sustainable gellator for enhanced oil recovery applications.

## Graphical abstract

In combination with a large group of various metal salts folic acid (FA) forms mechanically robust metallogels with rapid recovery and self‐healing properties. These versatile gel systems provide tunable stiffness, with up to 95% of stiffness restored within 1 min. Our work sheds light on FA as a high potential and universal gelator that is a relatively cheap and sustainable building block for soft materials with practical applications.

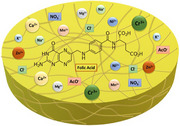

## 1 Introduction

Molecular gels are formed by self‐assembly of low molecular weight gelators in the presence of solvent molecules. As a branch of supramolecular chemistry, molecular gels constitute an attractive research topic in the development of functional smart soft materials. Their self‐assembly is driven by either one or a combination of several supramolecular interactions such as H‐bonding, π–π stacking, van der Waals forces, charge‐transfer interactions, or halogen bonding.^[^ ^1^, ^2^, ^3^ ^]^ Because of the weak nature of these non‐covalent interactions, molecular gels often provide facile reversibility when subjected to mechanical or other external stimuli. Therefore, they find potential applications in catalysis,^[^ ^4^, ^5^ ^]^ sensing,^[^ ^6^ ^]^ biological applications,^[^ ^7^, ^8^ ^]^ controlled release,^[^ ^9^ ^]^ and removing pollutants from water.^[^ ^10^ ^]^

Organic molecules with metal coordination sites allow metal‐ion‐induced gelation, commonly referred to as metallosupramolecular gels or simply metallogels. Metallogels combine the topological multiplicity of the metal–ligand interaction with multilevel hierarchical superstructures. The metal‐ligand interaction can lead to a primary assembly forming a one‐dimensional scaffold or can cross‐link the available one‐dimensional molecular assembly into a two‐dimensional or three‐dimensional complex network. Importantly, a metal cation can interact with an organic ligand through a coordinate covalent bond or a non‐covalent ion‐dipole interaction. Metallogels offer multiresponsive behavior by readily undergoing a gel ↔ sol transition. This can result in tunable stiffness, adaptivity, and orderliness of the gels.^[^ ^11^, ^12^, ^13^, ^14^, ^15^, ^16^, ^17^ ^]^ A wide range of stimuli such as temperature, oxidation‐reduction, pH, and electric and magnetic fields have been used to exploit the properties of coordinated metal ions.^[^ ^16^, ^18^, ^19^, ^20^ ^]^

In general, the ability to accept electrons and a wide range of coordination geometries of metal cations have led to the incorporation of transition metals and lanthanides into supramolecular metallogel networks.^[^ ^21^ ^]^ For example, it has been shown that pyridyl‐appended bis‐urea derivatives form metallogels in the presence of silver and copper and also allow in situ formation of nanoparticles.^[^ ^22^ ^]^ Functionalized derivatives of pyridine, bipyridine, and terpyridine have also been studied for anion/cation selective gelation, self‐healing properties, the formation of size‐ and shape‐selective nanoparticles in situ, and gelation‐induced luminescence. However, existing metallogels are usually specific to a particular metal ion or a counter anion.^[^ ^23^ ^]^

In the form of folate, folic acid (FA or vitamin B_9_) belongs to a class of water‐soluble B vitamins. FA is also a widely studied small‐molecular vector for drug delivery targeted at cancer cells.^[^ ^24^ ^]^ Despite extensive studies of the biological significance of FA, metal‐to‐FA complexation and related research on soft functional materials have been limited.^[^ ^25^, ^26^, ^27^, ^28^ ^]^ The presence of carboxylic acid groups and primary, secondary, and tertiary amines in the FA molecule make it quite a complex molecule capable of a variety of types of hydrogen bonding (as observed in its crystal structures)^[^ ^29^, ^30^, ^31^ ^]^ and metal‐coordination. Fazary et al. studied the complexation equilibria of FA with Fe(III), Fe(II), Ca(II), and Zn(II) in aqueous solutions. It was suggested that the metal ions coordinate with the carboxylates of the FA, making binary complexes. The stability constants of these complexes decreased in the order Fe(III)>Fe(II)>Zn(II)>Ca(II).^[^ ^25^ ^]^ Metal‐folate complexes can display high structural diversity, e.g., carboxylates of FA can function like a monodentate or a bidentate ligand binding to a single metal, or like a bridging bidentate ligand coordinating to two metals.^[^ ^27^, ^28^ ^]^

Although neat FA was studied for its gelation properties in a DMSO/water system,^[^ ^32^ ^]^ the resulting gel was mechanically weak, limiting its application. Therefore, several research attempts have been made to improve the mechanical properties of FA‐based gels. Song et al. prepared hybrid injectable gels by adding different amounts of agar to FA in a DMSO/water mixture. It was shown that agar molecules are involved in the process of self‐assembly via hydrogen bonds forming a denser 3D network, which improved the strength of the hybrid gel.^[^ ^33^ ^]^ In another study, the FA‐based supramolecular hydrogel was formed using Zn(NO_3_)_2_ and studied as a material for 3D printing.^[^ ^34^ ^]^ Embedding various metal salts inside molecular and polymeric gels can further fine‐tune their properties increasing the scope of diverse applications.

The structural organization of FA molecules in aqueous environments in the form of liquid crystals or organo‐/hydrogels has been previously studied.^[^ ^34^, ^35^, ^36^, ^37^, ^38^ ^]^ The FA or folate was found to form tetrameric structures through hydrogen bond interactions among pterin heterocycles (*Hoogsteen*‐bonded tetrad), assemblies analogous to those of the G‐tetrads (model comparison in Figure S1). These disk‐like arrays were further self‐assembled through π–π‐stacking to form tertiary columnar structures (similar to the well‐known G‐quadruplexes) where they are continuously rotated to each other, generating chiral columns. These columns further aggregate into fibrils, which entangle into a 3D fibrillar network of gels.^[^ ^39^, ^40^ ^]^

Despite the potential to create a diverse library of metallogels using FA, there exists no such systematic study to fully understand and eventually exploit this possibility. Herein, we provide an extensive investigation of FA‐metal salt gelation using a total of 33 metal salts complemented by a systematic evaluation of their mechanical properties. We show that FA acts as a supergelator that results in 17 metallogels at concentrations ≤ 0.2% (all % refers to w/v%, unless stated otherwise). More importantly, strong metallogels were obtained even at 0.07% in the presence of chromium(III) acetate. We show that the FA metallogels undergo rapid recovery, self‐healing, and thixotropy under shear rheology. We also demonstrate the macroscopic self‐healing of FA metallogels. Electron microscopy imaging revealed that all gels are composed of highly entangled fibrillar networks. Further insight into structure and morphology is provided using Fourier transformed infra‐red spectroscopy (FT‐IR), UV‐Vis spectroscopy, circular dichroism (CD), nuclear magnetic resonance spectroscopy (NMR), powder X‐ray diffraction (PXRD), and differential scanning calorimetry (DSC), suggesting new behavior of FA and a mechanism of self‐assembly in the presence of Cr(III)‐acetate.

The utilization of chromium‐based gels for enhanced oil recovery (EOR) represents a promising cost‐effective approach to increase the efficiency of oil mining. Chromium‐based polymeric gels are especially potent in enhancing oil recovery from unconventional reservoirs (e.g., with fractured formations). The gels act as conformance control agents that decrease water cuts in the oil fractions harvested and/or provide gas shutoffs. The process of employing chromium‐based gels is generally simple; gel precursors are prepared in solution and injected into the reservoir to modify the flow of fluid and to increase the displacement of oil.^[^ ^41^ ^]^ To optimize the gelation processes and to ensure optimal oil recovery operations, a choice of gel components, their concentrations, operating temperature, and environmental and geological conditions must be considered carefully. Alongside these, there are also concerns about environmental safety, acute to chronic toxicity, sustainability, and naturally the cost. A number of promising, mostly polymer‐based, metallogels have been studied with positive outputs in field tests.^[^ ^42^, ^43^, ^44^, ^45^, ^46^, ^47^ ^]^ For example, many oilfields in China have already undergone conformance control using chromium gels, e.g., the Huoshaoshan block in the Xinjiang oilfield. This block was developed by water injection in its early stage, but the poor physical properties of the original heterogenous formation led to the development of fractures resulting in low oil recovery. Field experiments with organic chromium gels were done in five groups of water injection wells, where the daily oil production of the 10 corresponding oil wells increased from 20.2 to 35.9 t, and the water content of the fluid produced decreased from 59.5% to 46.2%.^[^ ^48^ ^]^

In general polyacrylamide‐based chromium(III) gels are the most studied group of conformance control agents.^[^ ^42^, ^43^, ^44^, ^45^, ^46^, ^47^, ^49^, ^50^, ^51^ ^]^ However, drawbacks in their use are usually their low thermal stability and their sensitivity to high salinity.^[^ ^41^ ^]^ For these reasons, there is an ongoing search for advanced gel materials which can provide a broader range of applications in oil mining processes, while, maintaining overall safety and low cost. The initial assessment of the FA‐Cr(III)‐acetate metallogel encompasses the evaluation of gel formation in simulated seawater conditions, its aging, and scrutiny of thermal stability profiles. The FA‐Cr(III)‐acetate metallogel represents a promising candidate for EOR and shows good resistance to external stress. The use of sustainable and cheap FA provides metallogel stability, but also healable, thixotropic, and time‐controllable properties that can offer a new direction in EOR.

## 2 Results and Discussion

### 2.1 FA‐Based Metallogels

**Figure 1:**
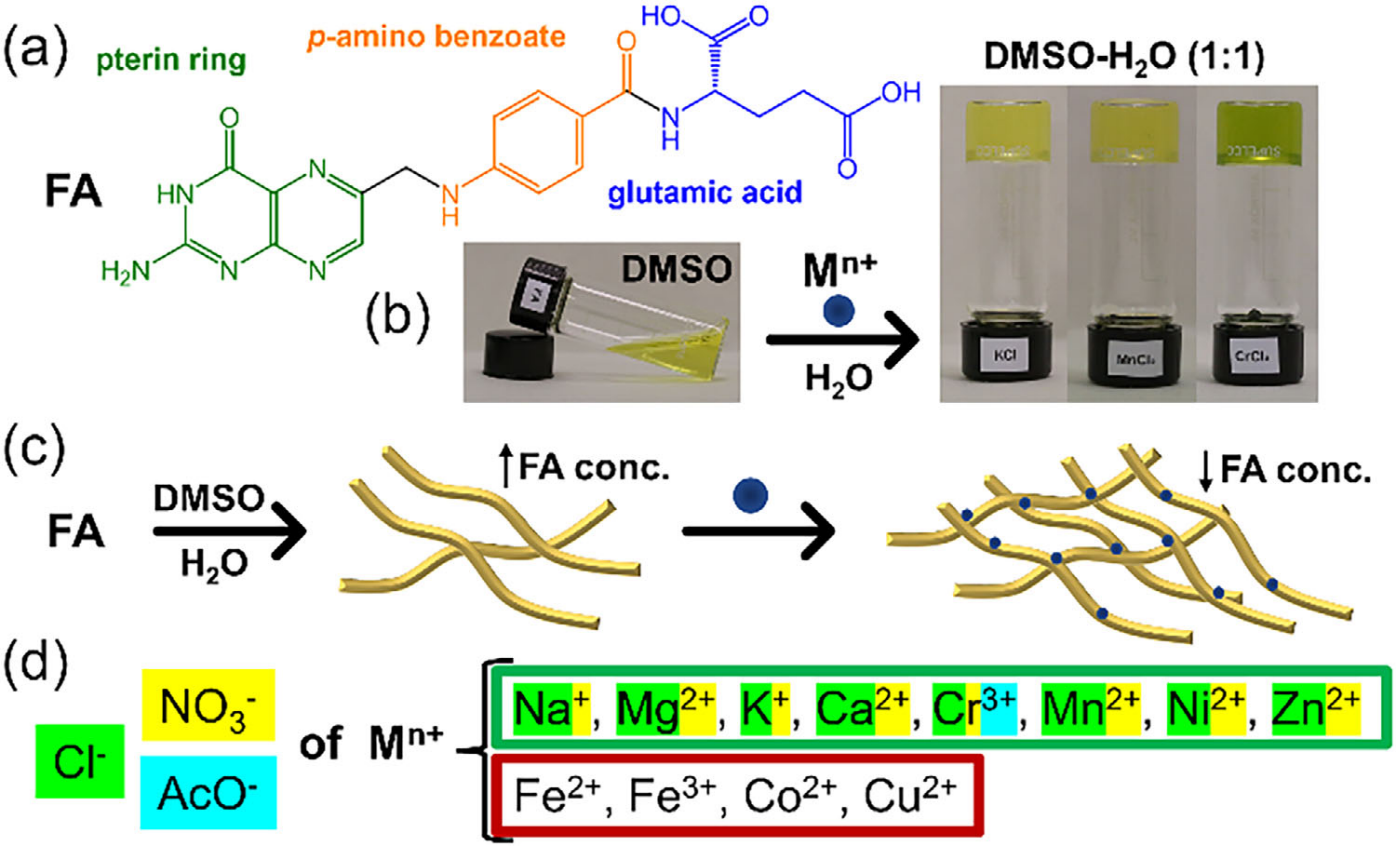
(a) Chemical structure of folic acid (FA). (b) Addition of aqueous solution of a metal salt to DMSO solution of FA promotes gelation as indicated by photographs showing resistance to flow upon inversion of the vial. (c) Self‐assembly of FA at high concentration and in the presence of metal salts at low concentrations in DMSO:H_2_O mixture (1:1). (d) Salts studied in this work: cations in the green frame promoted gelation, no gelation was observed using cations in the red frame. Green, yellow, and blue highlights refer to counter anions used for successful gelation.

We have tested the gelation of FA in a 1:1 (v/v) DMSO:H_2_O solvent system in the presence of monovalent, divalent, and trivalent metal salts. We have used 11 different metals as their chlorides, nitrates, and acetates, resulting in 33 different combinations (Table S1). Interestingly, we observed metallogelation in 17 systems leading to transparent yellowish or greenish‐yellow metallogels, depending on the metal ion under investigation (Figure 1 and Figure S2). All gels were formed at a concentration of 0.2%. Therefore, FA, together with metal salts, is referred to as a supergelator. Notably, the chromium(III) acetate gel formed at the extremely low concentration of 0.07%. Monovalent, divalent, and trivalent metals resulted in four (out of 6 tested), ten (of 13 tested), and three gels (of 6 tested), respectively. Of the 11 metal cations tested, eight metallogels were obtained with chlorides, and eight with nitrates, but only one metallogel was formed using an acetate salt (Figure 1).

The experiments were also carried out with representative salts of each valence group, NaCl, ZnCl_2_, CrCl_3_, and Cr(III)‐acetate in solvents with different H_2_O:DMSO ratios (0.4% of FA), 6:4, 7:3, 8:2, or 9:1. In all cases, weaker gels were formed in the 6:4 solvent system than in the case of 5:5. In the case of Cr(III)‐acetate, the gels were formed all the way up to the 9:1 ratio, and the time required for the gel to form was prolonged up to a week, but the strength of the gels decreased. In comparison, the Cr(III)‐acetate metallogel (0.2%) in H_2_O:DMSO 1:1 ratio formed a strong gel within 4–5 days (Figure S23). In general, the H_2_O:DMSO 1:1 (v/v) ratio was optimal, providing metallogels with the highest mechanical strengths as determined by the vial inversion test.

Moreover, we avoided using alkaline metal bases, e.g., KOH or NaOH to facilitate the formation of carboxylates.^[^ ^25^, ^34^ ^]^ By this approach, we aim to suppress the formation of strong coordination bonds between metal salts and carboxylates. It can play an important role when a mixture of salts is used, e.g., in advanced levels of oil mining, gelation is performed in the presence of seawater having excessive amounts of other metal salts, especially of Ca^2+^ or Mg^2+^, which can lead to unwanted partial precipitation of the gelator and thus decrease the effective gelator concentration resulting in gels with low mechanical strength and poor homogeneity.^[^ ^41^ ^]^ In addition, K^+^ and Na^+^ significantly affect the FA coordination self‐assembly (showing a preference for Na^+[^ ^35^, ^36^, ^37^ ^]^).

### 2.2 Rheological Properties of Metallogels

In general, metallogels have been studied for their unique rheological properties such as self‐healing and thixotropy.^[^ ^16^, ^52^, ^53^, ^54^, ^55^, ^56^ ^]^ We have used oscillatory shear rheological measurements to determine the mechanical properties of the FA metallogels. Accordingly, frequency‐sweep, time‐sweep, strain‐sweep, temperature‐sweep, and step‐strain experiments were performed. For rheological studies, 0.4% of premade gels were used.

**Figure 2:**
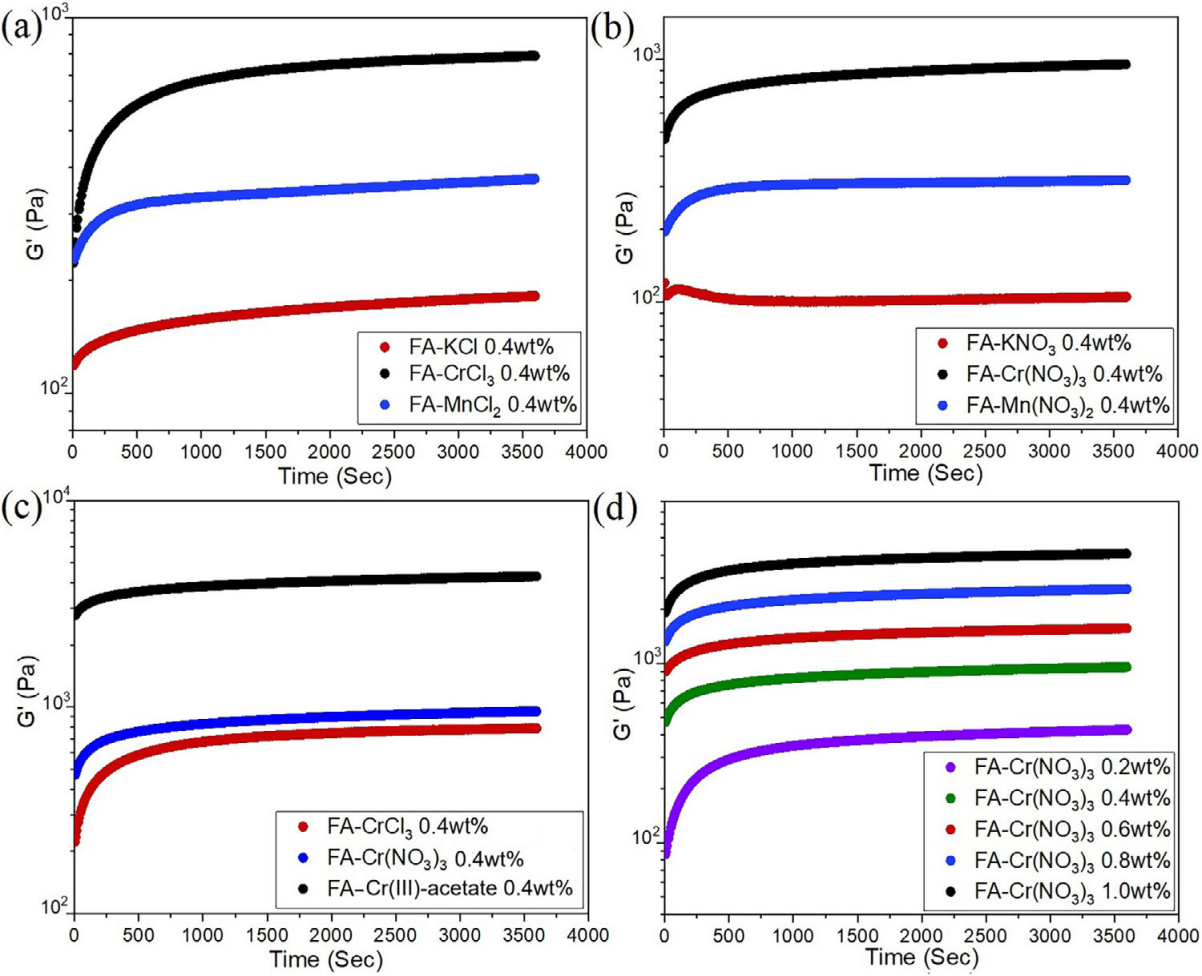
Time‐sweep experiments for 0.4% FA metallogels in DMSO:water (1:1) (a) comparison of chlorides of mono‐, di‐, and trivalent cations, (b) comparison of nitrates of mono‐, di‐, and trivalent cations, c) comparison of the effect of the anion for Cr3+, and d) the effect of the FA concentration in Cr(NO3)3 gel samples.

The time‐sweep experiment provides insights into the kinetics of gelation. The gelation behavior of the samples was monitored for up to 60 min after loading. Figure 2a,b show a comparison of the representative time‐sweep experiments of the 0.4% metallogels derived from chloride and nitrate salts of K^+^, Mn^2+^, and Cr^3+^. The elastic modulus G′ was found to be 0.1 kPa, 0.4 kPa, and 0.8 kPa, for FA‐KCl, FA‐MnCl_2_, and FA‐CrCl_3_, respectively (Figure 2a, b). Thus, in the case of chlorides, the gel stiffness follows the order: monovalent < divalent < trivalent cation. The trend was similar when the nitrate salts were used. Metallogels containing various divalent cations seem to have similar properties based on the comparison of their G′ values (Figure S3). Figure 2c,d show a comparison of the time‐sweep experiments for different anions of Cr^3+^ and variable concentration FA‐Cr(NO_3_)_3_ experiments, respectively. In general, the higher the concentration of components, the stiffer the metallogel. For a comparison of samples containing metal chlorides or nitrates, see Figure S3 in Supporting Information. Neat FA (without a metal salt) in DMSO:water (0.4%) could not be used for comparison with the metallogels as the sample does not form a gel (up to about 1.0%).

Figure S4 and Table S2 show results from frequency‐sweep experiments of selected monovalent, divalent, and trivalent chlorides, nitrates, and acetates. Typically, the storage modulus G′ is greater than the loss modulus G″ over an entire range of frequencies in the linear viscoelastic region (LVR).^[^ ^11^, ^57^ ^]^ In our experiments, G′ values are an order of magnitude higher than those of G″, suggesting that the materials under study are viscoelastic solids. For all of the metallogels studied, the frequency‐sweep results show that G′ and G″ are independent of the frequency. The storage modulus G´ decreased in the order of Cr^3+^>Mn^2+^>K^+^ for chlorides and nitrates. When the anions were varied in the case of the Cr^3+^ salt, the G′ followed the order FA‐Cr(III)‐acetate > FA‐Cr(NO_3_)_3 _> FA‐CrCl_3_ (Figure S4e). Notably, among all the metal salts tested, the Cr(III)‐acetate forms the strongest gels. The G′ of FA‐Cr(III)‐acetate metallogel (4 kPa) was ∼4 times higher than that of samples with Cr^3+^ nitrate or chloride salt. In another example comparing Cr(III)‐acetate with KCl metallogels, the G′ of Cr(III)‐acetate metallogel is even 40 times higher. The G′ values increase further with increasing concentration of the FA‐Cr(NO_3_)_3_ metallogel (Figure S4f), indicating the impact of an increased density of cross‐linkers on the mechanical properties of the metallogel. A reliable concentration study could not be performed with the FA‐Cr(III)‐acetate metallogels as they were found to be extremely strong and the samples slipped off the plates of the rheometer. Overall, the results suggest that the valency of the metal cations plays an important role in the stability and stiffness of the gel.

### 2.3 Self‐Healing and Recovery Studies

**Figure 3:**
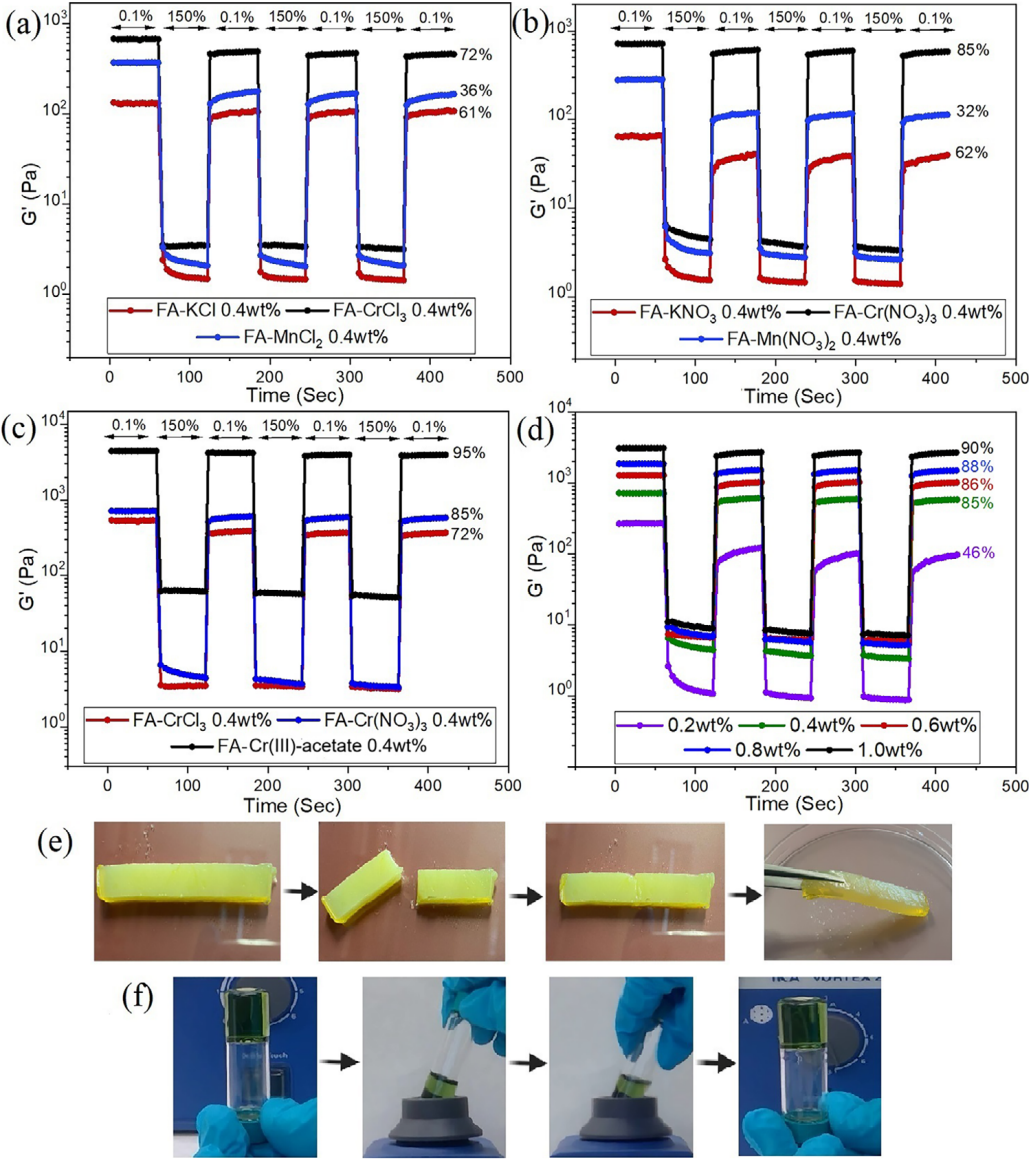
Step‐strain experiments showing the self‐healing properties of 0.4% DMSO:water (1:1) FA‐metallogels: (a) comparison of the self‐recovery properties of FA‐metallogels derived from chloride salts of mono‐, di‐, and trivalent cations, (b) comparison of nitrates of mono‐, di‐, and trivalent cations, (c) comparison of the self‐recovery properties of FA‐metallogels with different counter anions of Cr^3+^, (d) the effect of concentration on the self‐recovery of FA‐Cr(NO_3_)_3_ metallogel. (e) Photographs showing the macroscopic self‐healing of 1.0% DMSO:water (1:1) FA‐NaCl metallogel, and (f) photographs showing snapshots from Video S2 demonstrating the gel‐sol transition by the vortexing of the 0.2% DMSO:water (1:1) FA‐Cr(III)‐acetate metallogel.

Step‐strain experiments were performed to investigate the gel ↔ sol transition and self‐healing as the gel strength recovery (Figure 3). The gels showed a rapid decrease in G′ in response to an increased strain by turning into viscoelastic liquids. The application of increased strain over 60 s further breaks the structure as shown by the gradual decrease in the G′ values. Upon switching to a lower strain, the gels recover most of their original mechanical strength almost instantly, which reflects the rapid self‐healing properties of FA‐metallogels. Notably, in the step‐strain experiments, the recovery in the first cycle is not 100% of the original value. However, in the following cycles, they fully recover to the second G′ value. This is presumably due to a rearrangement of the gel structure in the first cycle and is typically also observed for other gels.^[^ ^52^, ^55^ ^]^ The process can be repeated for several cycles (Figure 3, herein for three cycles). Figure 3a shows the step‐strain rheological data for FA‐metallogels of KCl, MnCl_2_, and CrCl_3_. For FA‐metallogels with KCl and MnCl_2_, the equilibrium point was not reached compared to that of CrCl_3._ This suggests that the gels require a longer time (rest time) to recover completely (the recovery percentage for the first cycle is shown for each plot). Similar recovery behavior was also found for nitrates (Figure 3b). When three different salts of chromium were compared, the metallogel made of FA‐Cr(III)‐acetate showed the highest recovery of 95% in the first cycle (Figure 3c). In general, all of the FA‐metallogels with Cr^3+^ showed high recovery in their first cycle compared to other metal ions. The percentage recovery in the first cycle increased from 50% to 90% as the concentration was increased from 0.2% to 1.0% of FA‐Cr(NO_3_)_3_ metallogels (Figure 3d). Therefore, the results suggest that the extent of recovery depends positively on the strength of the gel. The strength of the gel depends on the metal cations and the concentration, and lesser on the counter anion used. Self‐healing was also observed with macroscopic gel samples. For these experiments, we prepared a free‐standing 1.0% of FA‐NaCl gel. The gel was cut into pieces which were subsequently re‐joined together. After 24 h, a uniform gel piece that could be lifted without any breakage was re‐formed (Figure 3e, Video S1). Furthermore, the immediate transition from sol to gel was observed when the FA‐Cr(III)‐acetate metallogel (0.2%) sample was subjected to intense mechanical perturbation using vortexing. The sol returned to the gel state immediately when vortexing was stopped (see Video S2). For more step‐strain experiments comparing metal chlorides and nitrates, see Figure S5 and Table S3 in Supporting Information. The results suggest that the gels are particularly mechanically robust, self‐healing, and thixotropic.

### 2.4 Temperature‐Sweep Experiment

**Figure 4:**
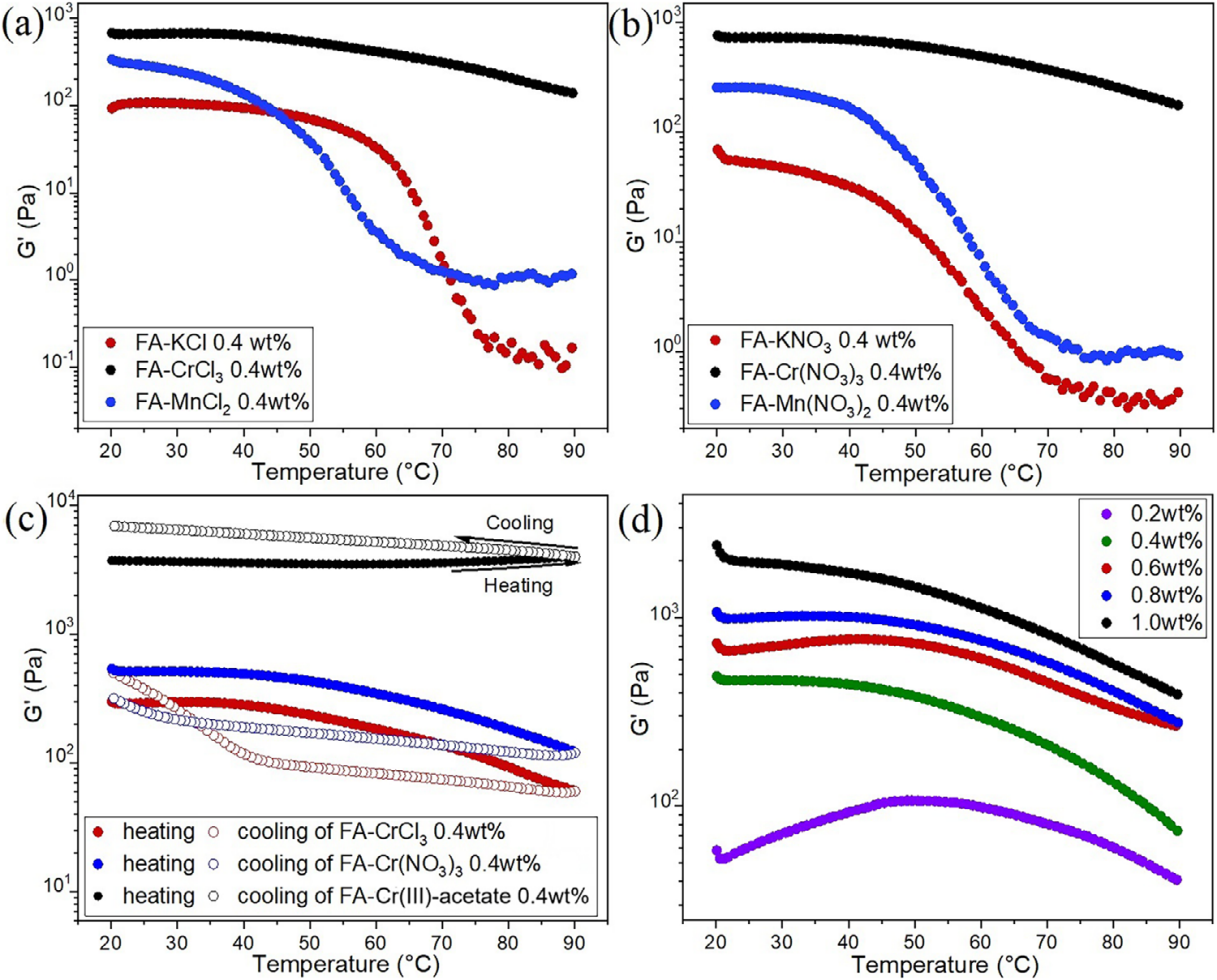
Temperature sweep rheological experiments of 0.4% DMSO:water (1:1) FA‐metallogels (a) comparison of the self‐recovery properties of FA‐metallogels derived from chlorides of mono‐, di‐, and trivalent cations, (b) comparison of the self‐recovery properties of FA‐metallogels derived from nitrates of mono‐, di‐, and trivalent cations, (c) effect of an anion on the thermal properties of FA‐Cr^3+^ metallogels, and (d) effect of concentration on gel melting properties of FA‐Cr(NO_3_)_3_ metallogels.

The gel→sol transition (melting profile) of metallogels was studied by temperature‐sweep rheological experiments (Figure 4a–d). The premade gels were heated (20°C–90°C) and cooled (90°C–20°C) and the moduli were followed. The gels showed rapid changes in their elastic moduli upon gel→sol transition, a typical characteristic of supramolecular gels.^[^ ^52^ ^]^ The elastic moduli of gels decreased by two orders of magnitude upon melting. Most of the gels showed the initiation of network melting (*T_onset_*) between 45°C and 55°C. Minima were recorded when the melting was completed (gel melting temperature *T_gel_*), which was between 55°C and 60°C, depending on the gel under study. Surprisingly, unlike other gels, no gel→sol transition was observed for any chromium gel (Figure 4c,d) in the temperature range of the sweep experiments. This implies that chromium metallogels only soften during heating in this temperature range and do not melt. In the cooling cycle, the gels with lower G′ values eventually reach the original G′ values (loop). Significantly, for the FA‐Cr(III)‐acetate sample, the G′ value decreased slightly as the temperature increased up to 60°C and then slightly increased (Figure 4c). Cooling the sample led to a further increase in the storage modulus. Between the beginning and the end of the experiment, ΔG′ was almost 2 kPa. This might imply that the gel network undergoes structural reorganization under heating leading to a stronger gel (but it could also correspond to a possible partial evaporation of solvent). For additional results comparing the effect of metal chlorides and nitrates on FA‐metallogels, see Figure S6 and Table S4 in Supporting Information.

### 2.5 Critical Strain and Yield Point

**Figure 5:**
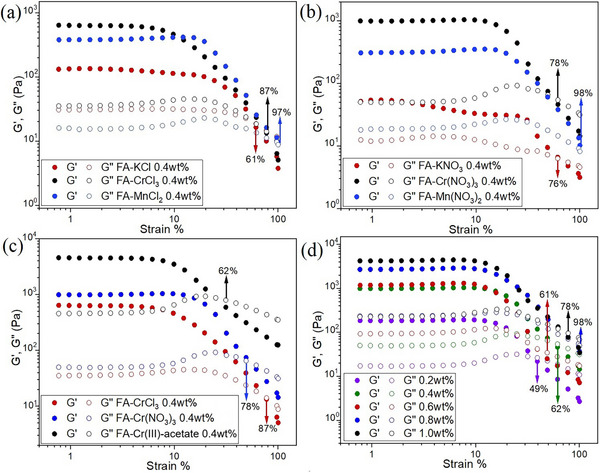
Strain‐sweep experiments for 0.4% DMSO:water (1:1) FA‐metallogels: (a) comparison of the critical strain of FA‐metallogels derived from chlorides of mono‐, di‐, and trivalent cations, (b) comparison of the critical strain of FA‐metallogels derived from nitrates of mono‐, di‐, and trivalent cations, (c) effect of an anion on the critical strain of Cr^3+^, and (d) effect of concentration on the critical strain of FA‐Cr(NO_3_)_3_ metallogels.

Beyond the linear‐viscosity region, G′ falls below G″ and the gel transforms into a liquid‐like form (sol). The rheological properties of soft materials display nonlinear behavior with a sudden decrease in their storage modulus above a certain strain level. This region is known as critical strain. The gel structure remains intact below the critical strain, but it is disrupted when the level of strain applied reaches above it. Thus, under high oscillatory stress, the solid‐like gel begins to flow. The term “yield stress” or “yield point” is the value of the crossover of G″ and G′, representing the stiffness.^[^ ^58^ ^]^ Yield stress and critical strain values are useful for understanding the linear viscoelastic regime of soft materials. For FA‐metallogels, the yield point values varied from 61% to 97%, depending on the cation and counter anion (Figure 5a–c). For the Cr(NO_3_)_3_ metallogel, the yield point values increased from 49% (for 0.2% gel) to 98% (for 1.0% gel), Figure 5d. For additional results comparing the effect of metal chlorides and nitrates on FA‐metallogels, see Figure S7 and Table S5 in Supporting Information.

Systematic rheological experiments were performed for all gels of the study (see Supporting Information, in Figure S8 we plot G′ at 1.194 rad/s vs. various metal salts). As could also be observed from their G′ values, the stability of the gels increases as their valence increases. The metallogels obtained with Na^+^, K^+^, Ni^2+^, and Mg^2+^ salts degraded after 3–4 weeks at room temperature, other gels were stable for months to a year. Interestingly, the FA‐Cr(III)‐acetate gel remained unchanged for almost three years.

### 2.6 Gelation Mechanism and Interactions

#### 2.6.1 Fourier Transform Infrared (FT‐IR) Spectroscopy

The FT‐IR spectra of samples of FA powder, Cr(III)‐acetate, FA gel (DMSO:water 1:1), and FA‐Cr(III)‐acetate metallogel (DMSO:water 1:1) were compared (Figure S9a). The FT‐IR spectrum of FA powder in the range of 3600–3000 cm^−1^ shows several vibrational bands: 3539 cm^−1^, represents the stretching vibration band of ─O─H in ─COOH groups; 3412 and 3319 cm^−1^, the antisymmetric and symmetric stretching vibrations (ν_as_, ν_s_) of ─N─H in ─NH_2_ groups; and ν_as_, ν_s_ of C─H at 2926, 2843 cm^−1^. In comparison, the broad shifted bands at 3380 and 3284 cm^−1^, present in both gel samples, suggest hydrogen bonding between FA molecules resulting in the formation of known pterin‐based tetrads.^[^ ^38^ ^]^ In comparison, the spectrum of the metallogel shows an additional vibration at 3660 cm^−1^ reflecting the stretching vibration of a free enolic ─OH group (Figure S9c)^[^ ^59^ ^]^ and suggesting the presence of a lactim tautomer (Scheme S1). The metallogel also shows very strong bands at 2987, 2972, and 2900 cm^−1^ corresponding to the O─H vibrations of carboxyls and C─H, and N─H stretching. It has been shown, that these signals can also provide information on the structural organization mediated through a complex system of hydrogen bonding, while the peaks around 3012 cm^−1^ are attributed to the organization in disc‐like arrays (signals at 3150 cm^−1^ would correspond to a ribbon‐like pattern, but are missing in our case).^[^ ^41^, ^60^, ^61^ ^]^ At the lower wavenumbers, the absorption bands of C═O stretching of the ─COOH or ─CONH─ groups of FA at 1690 and 1620 cm^−1^, respectively, and the ─NH─ of ─CONH─ at 1602 cm^−1^ merged into a wide absorption band at 1647 cm^−1^ in both gel samples indicating the involvement of these groups in hydrogen bonding. Comparing the spectra of both gels, the common strong broad band at 1647 cm^−1^ belongs to the C═O stretching of CONH, COOH, or a conjugated carboxyl indicating the involvement of these groups in hydrogen bonding or adjacent metal coordination. The characteristic absorption band of the phenyl ring at 1480 cm^−1^ in FA powder, shifts to 1400 cm^−1^ in gels. There are also several differences between the gels: the O─H bending band is larger for the metallogel sample, and the metallogel shows an additional aromatic amine C─N vibration at 1250 cm^−1^ and an alcohol C─O stretching vibration at 1050 cm^−1^. In both gels, we can observe the strong S═O stretching vibrations at 1012 and 951 cm^−1^ of the DMSO molecules.

Based on the differences observed between the FA‐gel and metallogel we speculate that FA undergoes a partial chemical transformation in the presence of chromium salt which subsequently directs the 3D self‐assembly into the complex network of metallogel. The pH of FA‐Cr(III)‐acetate gel is 6, which suggests that the FA is mostly present in the form of folate. Previous studies have also shown tautomerism of folate manifested in aqueous solution in the form of an equilibrium mixture of N3 and N1‐lactams (keto‐tautomers) rather than as a lactim (enol‐tautomer) (Scheme S1,^[^ ^62^ ^]^ electrostatic potential map of N3‐lactam in Figure S21). The addition of Cr(III)‐acetate can lead subsequently, to establishing a new equilibrium among various forms, e.g., the presence of acetate can stabilize the hydrogen bonding of lactam but also lactim forms,^[^ ^63^ ^]^ and the lactam tetrads can bind the Cr(III) ion through ion‐dipole interactions (Scheme S1). Overall, several chemical forms of FA seem to be present in the complex structure of the FA‐Cr(III)‐acetate metallogel at the same time, which makes it rather difficult to describe in detail using IR spectroscopy. Nevertheless, this method provides us with valuable initial information on the gel structure that can be further evaluated by a combination of other analytical techniques.

Not only the structure of FA but also the form of chromium(III) acetate plays an important role in the gelation process. Chromium(III) acetate basic is a trimeric cluster, [Cr_3_O(O_2_CCH_3_)_6_(OH_2_)_3_](O_2_CCH_3_), with a well‐defined Cr_3_O core stabilized by acetate ligands and a central μ_3_‐oxo bridge. Previous reports indicate that the Cr_3_O cluster exhibits stability in an aqueous environment, although ligand exchange can occur depending on the coordination environment and pH conditions.^[^ ^64^, ^65^ ^]^ In the IR spectrum of neat chromium(III) acetate basic, distinct vibrational modes are observed in the region of 500–700 cm^−1^ corresponding to the Cr─O─Cr core (Cr_3_O): band at 544 cm^−1^ attributed to Cr─O─Cr asymmetric stretching, 620 cm^−1^ band assigned to Cr─O─Cr bending vibrations, and band at 654 cm^−1^ referring to Cr─O stretching modes (Figure S9d).^[^ ^66^ ^]^ The gel sample exhibited a broad band in this spectral region with one sharper band at about 708 cm^−1^ consistent with vibrational modes of the Cr_3_O core (Figure S9d). The broadening is caused by non‐covalent and coordination interactions of the Cr_3_O core with FA functionalities assuming that under the gelation conditions, the acetates are to some extent replaced by FA carboxylates.^[^ ^65^ ^]^ At the same time, having a complex equilibrium reaction going on here, we consider that some amount of *tris*‐chromium(III) cluster decomposes into *mono*‐chromium(III) cations which can be incorporated within pterin tetrads through ion‐dipole interactions, stabilizing overall the metallogel network. Although the ion‐dipole interaction is a weaker non‐covalent force, in its cooperativity it is exceptionally robust and supports the formation of strong gels even in the presence of a multi‐component mixture such as seawater (experiments shown below).

#### 2.6.2 CD Spectroscopy

**Figure 6:**
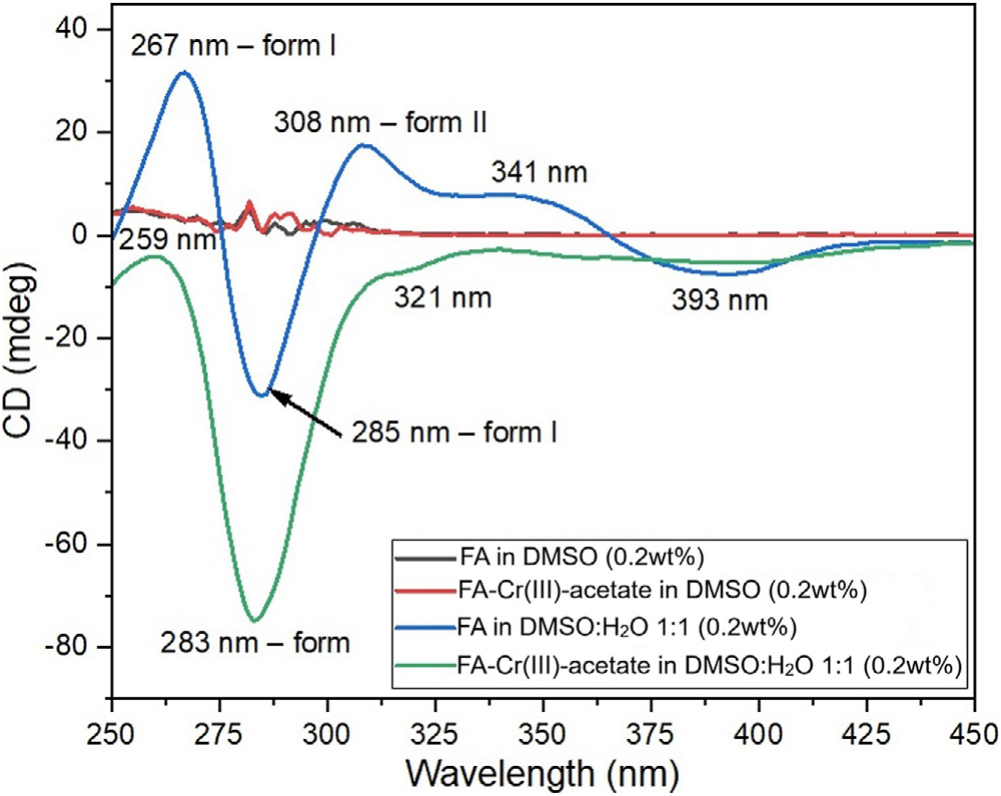
Circular dichroism spectra of FA and FA and Cr(III)‐acetate in DMSO or DMSO:H_2_O mixture showing the presence of two forms of supramolecular aggregates.

The CD spectra of FA in DMSO with and without metal salts show no chiral self‐assemblies at the given concentrations of the components; any molecular chirality is silent in the CD spectra. On the other hand, the addition of water to the DMSO solution induces significant changes in the spectra because a supramolecular chiral self‐assembly is formed (Figure 6). In the case of neat FA, the spectral shape is characterized by a broad positive band with a maximum at 267 nm, followed by a broad negative band at 285 nm, which further continues to a positive band at 308 nm, complemented by a lower very broad positive band at 341 nm and a similar but negative band at 393 nm. The addition of chromium(III) acetate leads to significant changes in the spectrum and the disappearance of several of the bands characteristic of neat FA. The intensity of the negative band at 283 nm significantly increases and is complemented by a weak band at 321 nm, after which the spectrum approximates to zero, but still has a very broad negative band at around 390 nm.

These results can be compared with those previously observed and elucidated by Gottarelli et al.,^[^ ^37^ ^]^ where the folate has been found to form two different hexagonal columnar mesophases, I and II, in the presence of Na^+^ or K^+^. This was related to the formation of *Hoogsteen*‐bonded tetramers and their pterin‐mediated π–π stacking into larger columnar helical aggregates. Form I is characterized by a positive band at about 270 nm and a negative one at 290 nm, whereas form II has a positive band at about 310 nm. The difference between the two phases is minimal, originating mainly in the tightness of stacking. Form I shows a greater aromatic stacking distance than form II, which is tighter. The attractive interaction between quadruplexes was also found to be significantly lower in pure water and increased with increasing concentrations of salts (the effect of Na^+^ is greater than that of K^+^). Both forms show that the glutamate side‐chain is rather mobile in the structure, not directly affecting the chiral aggregation.^[^ ^37^ ^]^

Similarly, in our samples, we can also observe the presence of both forms, I and II. The FA gel sample shows the presence of form I, having positive bands at 267 and 285 nm, and form II with a band at 308 nm. In contrast, the addition of a Cr^3+^ salt leads to the appearance of a very strong signal at 283 nm, which can be explained by the presence of only form I. Interestingly, the measurements were also carried out at various time periods. Whereas at 15 min the FA gel sample shows the presence of only form I, at 45 min form II also appears (after this period the composition of the sample remains the same) (Figure S10). In contrast, the spectrum of the FA‐Cr(NO_3_)_3_‐metallogel sample does not change over time after the first measurement at 15 min. These findings can be referred to the previously observed trend where the tightness and length of stacked quadruplexes decreased with increasing size of the cation, Na^+^ versus K^+^,^[^ ^37^ ^]^ where Cr^3+^, being an even larger cation, can lead selectively to the formation of the loosen form I. Nevertheless, the tightness of the interaction among quadruplexes does not correlate directly with the strength of metallogels, and other structural and physicochemical factors will also be considered. Another interpretation of the presence of form I is correlated with the presence of *tris*‐chromium(III) acetate cluster. Being a metal nod too large to be effectively intercalated within the tetrads, the cluster preferably coordinates with carboxylates of the side chain. This can similarly disturb the tight packing of pterin tetrads by “pulling” the FA molecules apart. These together with the hydrogen‐bonded and/or coordinated molecules of solvents contribute to tetrad's distortion and increased distancing.

#### 2.6.3 UV‐Visible Spectroscopy

**Figure 7:**
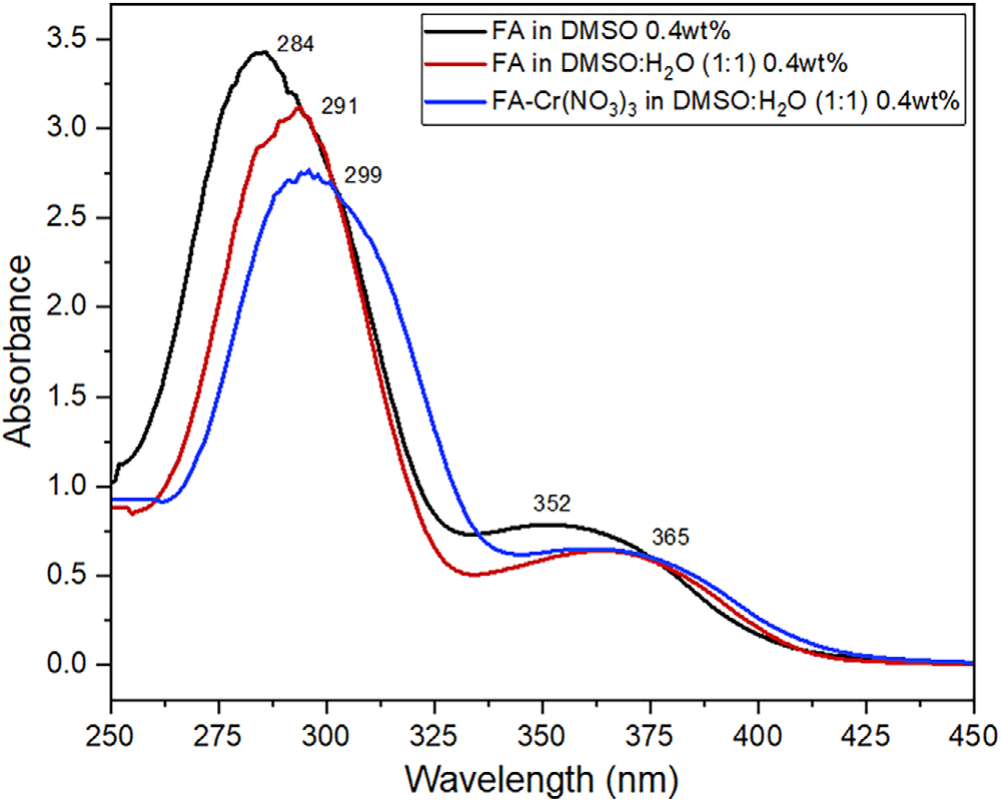
UV‐Vis spectra of FA powder in DMSO, FA gel, and FA‐Cr(NO_3_)_3_ gel in DMSO:water (1:1) (all experiments were measured at 0.4 wt% and room temperature).

UV‐Vis spectra of FA in DMSO, and FA‐gel and FA‐Cr(NO_3_)_3_‐metallogel in DMSO:H_2_O (1:1) were measured at 0.4 wt% and at room temperature (Figure 7). The absorption bands of the pterin ring in the FA‐DMSO solution appear at 284 and 352 nm and are attributed to π⟶π* and n⟶π* transitions, respectively. The corresponding absorption bands of the pterin ring in the FA gel show the bathochromic shift of 7 and 10 nm, respectively, which indicates that FA molecules self‐assemble in the DMSO:H_2_O solvent mixture through π–π stacking interactions in a J‐type aggregate. J‐aggregates are represented by an “angle of slippage” larger than 32° (the angle between the line of centers of a column of molecules and the long axis of any one of the parallel molecules). Thus, the heterocycles stacked on top of each other in tetrads are rotated to each other forming a helical supramolecular structure. This can be further supported by decreasing the absorption intensity of the peak at 291 nm. In the case of FA‐Cr(NO_3_)_3_‐metallogel, the absorption bands of pterin show an additional 8 nm bathochromic shift as well as an additional lowering of the absorbance when compared to FA gel, which is connected to the effect of electrostatic ion‐dipole interactions of the metal to the carbonyls of the pterin tetrad and additional aggregation in this complex gel network.

#### 2.6.4 ^1^H‐NMR Spectroscopy and Variable Temperature NMR

NMR spectroscopy experiments have been used to reveal the non‐covalent interactions involved in gel formation and its dynamics. However, the NMR spectroscopic characterization of certain metal complexes is a challenging task because some of them are paramagnetic.^[^ ^67^, ^68^ ^]^ Therefore, out of all of the samples studied, the FA‐KCl, FA‐NaCl, FA‐ZnCl_2_, and FA‐Zn(NO_3_)_2_ gel samples were selected for the NMR studies as diamagnetic species, and one representative of the paramagnetic group of metals, Cr(III)‐acetate, was included.

**Figure 8:**
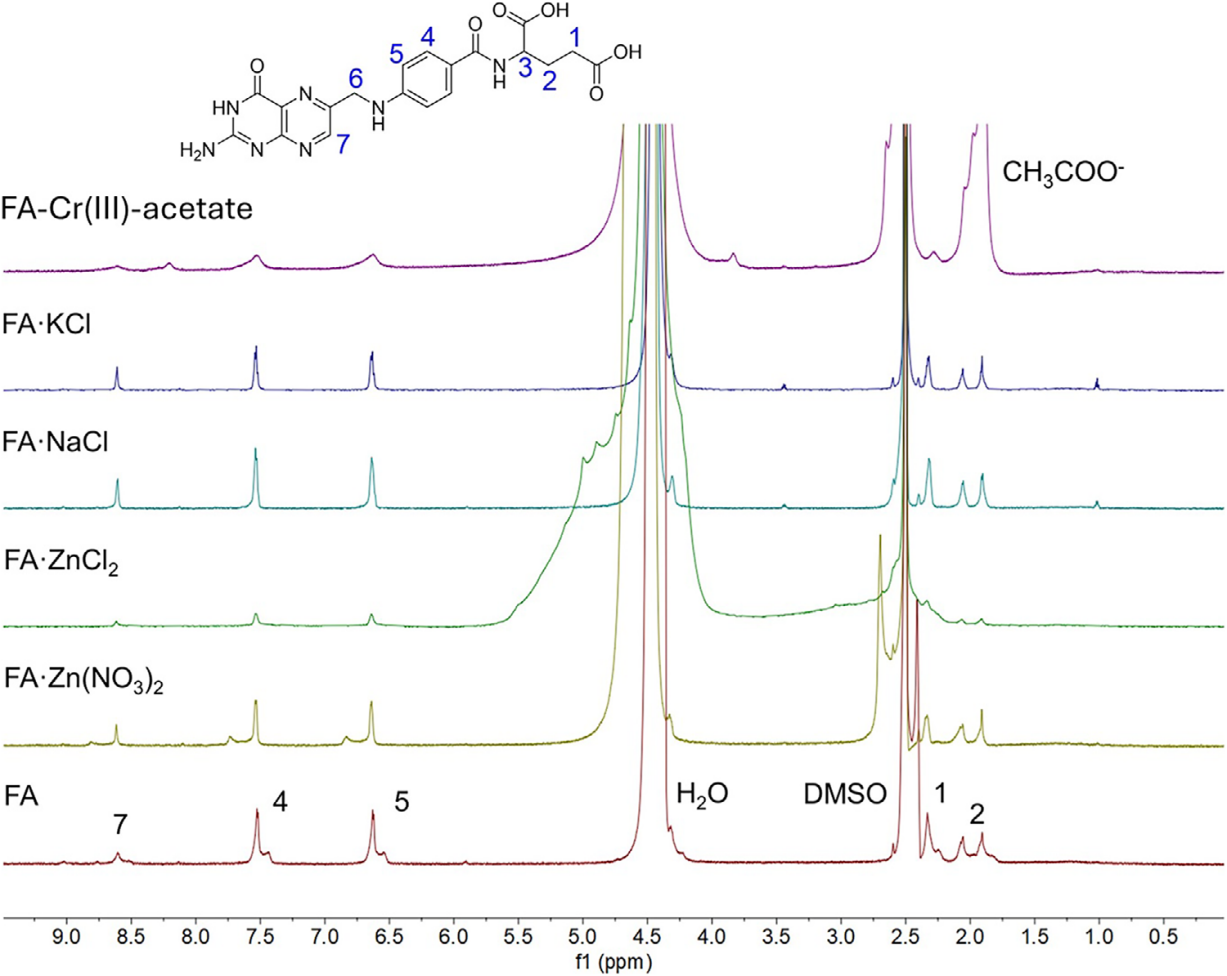
^1^H NMR of folic acid (FA) and its metallogels (0.4 wt% of FA and metal salt:FA 1:1) in DMSO‐*d*_6_:D_2_O 1:1 (298.2 K, 700 MHz).

Initially, we measured the ^1^H NMR spectrum of the FA sample in DMSO‐*d*_6_ and in the mixture DMSO‐*d*_6_:D_2_O 1:1. Whereas the FA in DMSO‐*d*_6_ provides a single set of ^1^H NMR signals (Figure S11), the FA solution in the mixture DMSO‐*d*_6_:D_2_O 1:1 provides two sets of broader ^1^H signals, suggesting the involvement of water molecules in the FA self‐assembly, possibly via hydrogen bonding (Figure 8). The very broad ^1^H signals for carboxylic acid groups and ─NH_2_ can also suggest the formation of intermolecular H‐bonding. Moreover, the absence of a signal for hydroxyl protons in DMSO‐*d_6_* suggests that the lactim tautomer is not significantly represented in the liquid sample. The overlapping sets of signals, a major and a minor set in approximately 4:1 ratio have a chemical shift difference Δ*δ* = 0.08 ppm, where the minor set is at lower frequencies. Their presence can have several explanations, related to the previous structural studies. The major signal set can represent the formation of hydrogen‐bonded tetrads with the minor set representing their short oligomeric π–π‐stacked aggregates or even a ribbon‐like assembly in solution.^[^ ^38^, ^60^, ^61^ ^]^ When analyzing the NMR data we should also keep in mind that large(r) polymeric aggregates are not visible in a regular solution‐state ^1^H NMR spectrum. Thus, we cannot see the pivotal gel polymeric framework but only small fragments of its possible building blocks. Figure 8 further shows a comparison of the spectrum of neat FA with spectra of various FA metallogels at 0.4 wt% in DMSO‐*d*_6_:D_2_O 1:1 mixture. Several differences can be observed in the ^1^H NMR spectra. The minor second set of signals observed in the neat sample of FA disappears in all of the metallogels studied, likely because of metal‐induced aggregation into larger aggregates. In the Zn(NO_3_)_2_ sample, next to the familiar major signal set, a new second set of signals appears at higher frequencies with Δ*δ* = 0.20 ppm. This may represent tetrads carrying Zn^2+^. ZnCl_2_, NaCl, and KCl samples provide only a single set of signals similar to the major signals of neat FA representing free tetrads. In general, chlorides form stronger gels than nitrates, which could be translated here into a more effective polymerization of gel fibers using chlorides rather than nitrate. Finally, the addition of paramagnetic Cr(III)‐acetate leads to a significant broadening of all FA signals and the appearance of a new signal at 8.21 ppm (this may originate from a signal of coordinated species—a paramagnetic shift of some proton signal).

In order to suppress the effect of water on the gelation of FA and to see any possible changes in the chemical shifts of FA induced by interactions with metal salts, we performed an ^1^H NMR titration experiment of FA DMSO‐*d*_6_ solution with Zn(NO_3_)_2_ (Figure S12). The FA DMSO‐*d*_6_ solution gives only one set of NMR signals and there are no changes in the spectra upon the addition of metal salt, except for a broadening of the ─NH_c_‐signal. Finally, variable temperature (VT, from 298.2 K to 348.2 K) ^1^H NMR experiments were carried out to follow on sol ↔ gel transitions of FA gel (0.2 wt%) and FA metallogels (0.8 wt% of FA; using Zn(NO_3_)_2_, ZnCl_2_, NaCl, KCl, and Cr(III)‐acetate in 1:1 ratio with FA) prepared in a DMSO‐*d_6_*:D_2_O 1:1 mixture (Figure S13‐18). In all cases a significant increase in the signal intensity was observed with increasing temperature of the sample, suggesting a gel → sol transition (melting of the gel leading to an increased concentration of small assemblies of FA). As in the temperature‐sweep rheologic experiments, gradually cooling the samples inside the probe to 298.2 K led to a significant decrease in the intensity of the NMR signals representing the sol → gel transition (gel recovery).

Even though we could not observe significant changes in the ^1^H NMR spectra suggesting effective metal‐FA interaction, we could follow the presence of smaller FA‐aggregates. We can assume that the presence of metal cations leads to the swift formation of larger polymeric aggregates which are not directly visible using liquid‐state NMR.

#### 2.6.5 Powder X‐Ray Diffraction (PXRD)

To gain further insight into the solid‐state properties of the metallogels, PXRD measurements were performed on dried samples. In general, the samples do not show any signs of crystallinity from the PXRD pattern (e.g., FA‐Cr(III)‐acetate in Figure S19a), suggesting that the self‐assembled aggregates in metallogels are too small (the reported size at the same concentration of FA in the presence of K^+^ or Na^+^ is 9 stacked tetramers)^[^ ^37^ ^]^ or too few to be detected by PXRD (but aggregates are apparently present as shown by the more sensitive CD spectroscopy). Also, it is well documented in the literature that thixotropic gels display an amorphous nature in their XRD pattern.^[^ ^69^ ^]^

#### 2.6.6 Differential Scanning Calorimetry (DSC)

For DSC studies, dry or wet samples were weighed into a pan and measured. In general, the thermal responsiveness of the gels is influenced by their structural features, e.g., nanofiber structure, and/or their crosslinking in the gels. The supramolecular gel network is therefore quite sensitive to temperature, particularly near the critical gelation temperature. For example, the DSC curve of the FA‐Mg(NO_3_)_2_ sample (Figure S19b) shows endothermic peaks between 45°C and 60°C that account for the gel‐solution phase transitions. On the other hand, the FA‐Cr(NO_3_)_3_ (Figure S12b) and the FA‐Cr(III)‐acetate (Figure S12c) gel samples do not follow the same trend as they show only negligible decrease in heat flow over the temperature range. The gels do not melt (only soften), which is in agreement with the results of rheological temperature‐sweep experiments and can also be observed from the VT ^1^H NMR spectrum.

#### 2.6.7 Electron Microscopy (EM)

**Figure 9:**
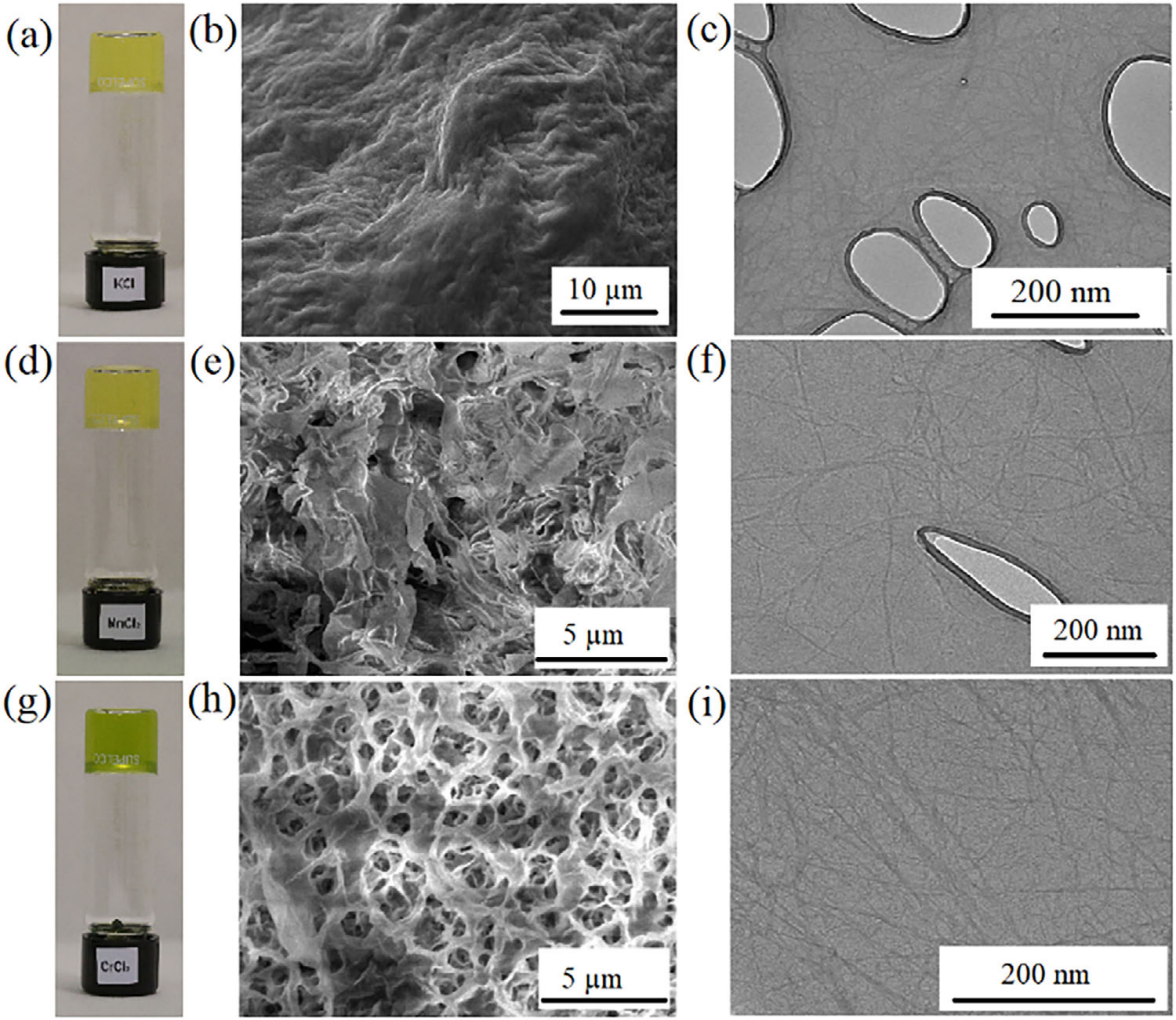
(a) Photograph of 0.2 wt% FA‐KCl metallogel in DMSO:water mixture (1:1), (b) SEM image of freeze dried FA‐KCl metallogel , (c) TEM image of FA‐KCl metallogel. (d) Photograph of 0.2 wt% FA‐MnCl_2_ metallogel in DMSO:water mixture (1:1), (e) SEM image of freeze dried FA‐MnCl_2_ metallogel , (f) TEM image of FA‐MnCl_2_ metallogel. (g) Photograph of 0.2 wt% FA‐CrCl_3_ metallogel in DMSO:water mixture (1:1), (h) SEM image of freeze dried FA‐CrCl_3_ metallogel, (i) TEM image of FA‐CrCl_3_ metallogel.

To investigate the structure of the supramolecular assemblies, we performed EM imaging of all of the gels studied. The TEM images of all 17 metallogels revealed the presence of highly entangled fibrillar networks. The diameter of the fiber varied from 5 to 50 nm. The type and size of the fiber were similar for all of the gels studied. However, in some cases, a strong film formed as the gels dried and prevented the extraction of finer details. For SEM imaging, the gels were freeze‐dried before sputtering and imaging. The SEM images of freeze‐dried gels also displayed highly entangled networks. Figure 9 shows representative TEM and SEM images of the FA‐KCl, FA‐MnCl_2_, and FA‐CrCl_3_ metallogels. Figure S20 shows the remaining TEM and SEM images of the metallogels obtained.

#### 2.6.8 Enhanced Oil Recovery Using Metallogels

**Figure 10:**
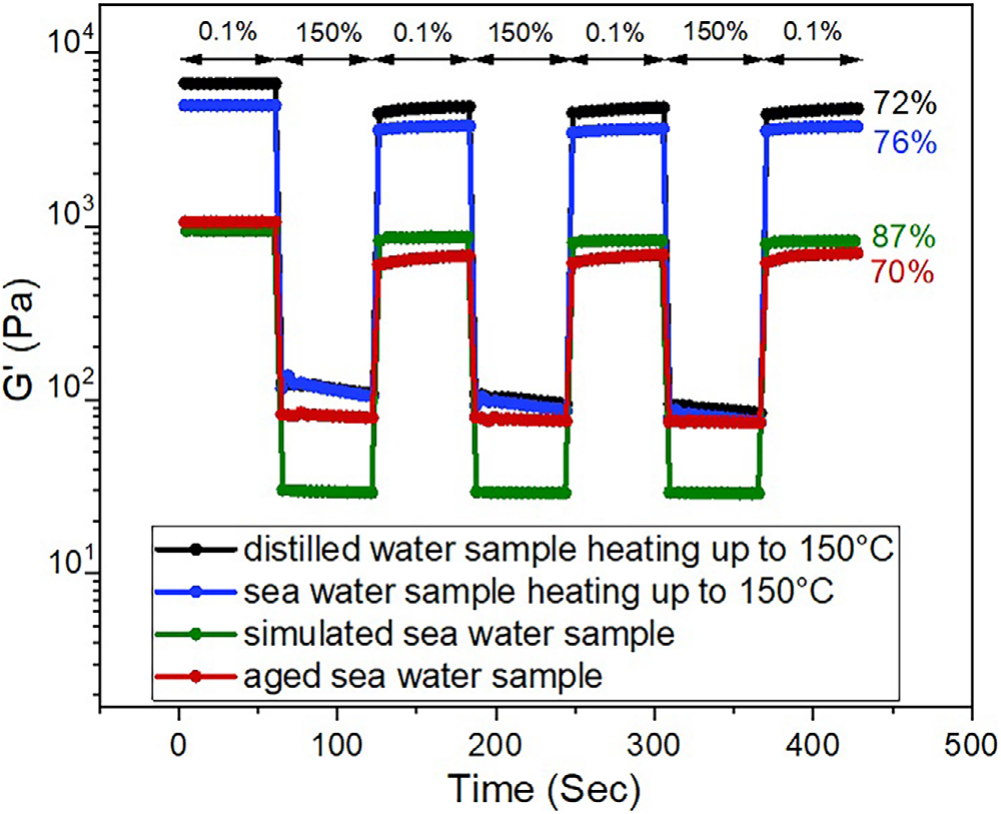
Step‐strain experiments showing the self‐healing properties of FA‐Cr(III)‐acetate metallogels (0.2 wt%) under various conditions mimicking those in field tests, i.e., thermal stress at 150°C, seawater environment, and aging effect.

To explore the potential applications of the FA‐Cr(III)‐acetate gel (0.2%), we carried out proof‐of‐concept experiments for oil recovery. Accordingly, we carried out experiments on the effect of simulated seawater (a mixture of NaCl, MgSO_4_, CaCl_2_, and KCl in distilled water (with overall salt concentration 34 g/L)) (Table S7 and S8) or saturated brine solution (335 g/L) on gel formation and stability, changing reagents’ concentration and ratio (simulating possible dilution in oil reservoir) and following the rate of gel formation and its strength at ambient temperature or 150°C (mimicking conditions of high‐temperature oil reservoirs) (Table S6), following on the effect of thermal stress (heating the samples for 12 h at 100°C, 120°C, 150°C, 200°C, and 250°C), or aging of the samples for one year (Figure 10 and S22, Table S7).

The gels can be formed at ambient temperature at various concentrations down to 0.07%. The gels are also formed at various FA to Cr(III)‐acetate ratios, i.e., from 1:1 down to 100:1 ratio. At 150°C, strong gels are slowly formed at 0.4% concentration (FA:Cr(III)‐acetate 1:1) within 24 h and are further stiffening for up to 7 days of the study (Table S6).

Moreover, the effect of thermal stress (heating the 0.2% FA:Cr(III)‐acetate 1:1 samples for 12 h at 100°C, 120°C, 150°C, 200°C, and 250°C) and aging the samples for one year (0.2% FA FA:Cr(III)‐acetate 1:1 in water:DMSO or seawater:DMSO 1:1) were followed by rheological measurements. Frequency‐sweep rheological experiments on these samples showed that the materials remained viscoelastic solids upon aging or thermal treatment up to 200°C (Figure S22f,g). Time‐sweep experiments showed that the gel strength of the samples remained constant as follows for 60 min (Figure S22b,c). Temperature‐sweep experiments further confirmed the good thermal stability of the gels within the 20°C–80°C range (Figure S22d,e). The sudden drop in gel strength at 250°C is likely caused by the thermal decomposition of FA.^[^ ^70^, ^71^, ^72^ ^]^ whereas at the lower temperatures studied, i.e., 100°C, 120°C, 150°C, and 200°C, the gels increased in strength as followed by G′ values (Table S7). Considering that gelation is a dynamic process driven by temperature‐dependent kinetic factors, the higher the temperature, the faster the rearrangement towards thermodynamically favored rearrangement of the gel structure resulting in a stronger gel network. Step‐strain and strain‐sweep experiments using the thermally treated samples showed critical strain values around 80% after one cycle, even in the presence of seawater (Figure 10, S22a,h,i,j).

The effect of simulated seawater on gel formation was studied at ambient temperature and at 150°C (Table S8). Rigid gel (0.4 wt% FA in seawater:DMSO mixture 1:1) can be formed at ambient conditions after 7 days. The flowing gel can be formed at 150°C under the same conditions and time. Excellent stability of FA:Cr(III)‐acetate metallogel (0.4 wt%, 1:1 ratio in water:DMSO 1:1) was further confirmed in the presence of saturated brine solution at room temperature but also under heating for one week at 150°C.

Overall, the results show that the FA‐Cr(III)‐acetate gels are mechanically robust, thermally stable, and maintain their self‐healing and thixotropic properties. Moreover, the stability of the gels increases with an increasing temperature up to 200°C. The gels are not sensitive to the presence of other cations/anions in the solvent system, but quite the opposite, their mechanical strength increases in the presence of seawater (and further upon heating) (Table S7).

## 3 Conclusions

In this work, we show that the incorporation of quite a large group of various metal salts via the hierarchical self‐assembly of natural FA results in supramolecular metallogels with a robust mechanical elastic modulus at concentrations below 0.2%. Combinations of FA with chlorides, nitrates, and acetates of Na^+^, K^+^, Mg^2+^, Ca^2+^, Mn^2+^, Co^2+^, Ni^2+^, Cu^2+^, Zn^2+^, Fe^3+^, and Cr^3+^ were investigated, out of which 17 supragels were prepared and thoroughly studied for their rheological behavior, morphology, and structure using SEM, TEM, ^1^H‐NMR, FTIR, CD, UV‐Vis, PXRD, and DSC. Rheological studies proved that the addition of a metal ion to FA significantly increases the strength of the gels. Comparing their storage modulus values G′, the strength of the gels decreases in a row for chlorides and nitrates in the same way: Cr^3+^ > Ca^2+^ > Zn^2+^ > Mn^2+^ > Mg^2+^ > Ni^2+^ > Na^+^ > K^+^. The FA‐based metallogels studied show that their strengths are also influenced by the counter anion, chlorides mostly resulting in stronger gels than nitrates (with the exception of Cr^3+^). In the case of chromium(III)‐based systems it seems that the effect of the cation represents a dominant factor in the strength of the gels with, exceptionally, the strongest gel formed using Cr(III)‐acetate (it being also the only acetate‐based metallogel found in this work). The robustness of FA gelation with metals is outstanding in its non‐selective character, as the FA can form strong gels with 17 metal salts in a relatively broad range of concentrations and conditions.

**Figure 11:**
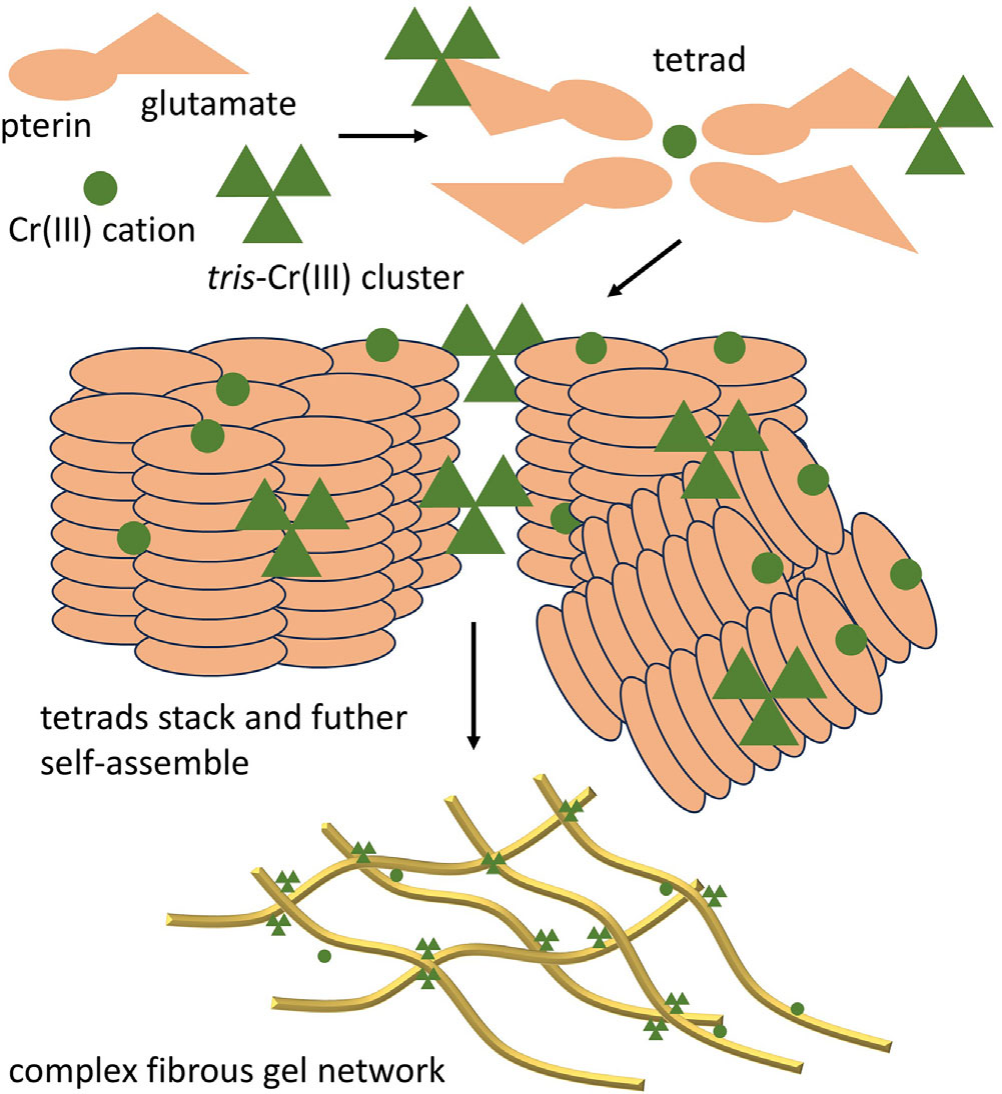
Mode of self‐assembly of FA in the presence of Cr(III)‐acetate in DMSO:water mixture.

Given the large group of variable metal salts tested, monovalent, divalent, or trivalent alkaline, alkaline earth, or transition metal salts, their morphology varies (as seen by SEM and TEM) and their (supra)molecular structure can also vary along with various mechanical properties, as established by rheological studies. Of the group of metallogels, we have closely investigated the molecular structure of the strongest, the most stable, and for us currently, the most interesting metallogel derived from Cr(III)‐acetate. Using a combination of analytical techniques, we found that FA follows a general trend of self‐assembly into tetrads and their stacking into small columnar aggregates. The Cr^3+^ can be partly positioned in the center of these tetrads but can also decorate the outside of the aggregates that arise and lead to their additional interlinking. We hypothesize that the pivotal network forming π–π and Cr(III)‐mediated columnar stacks of tetrads are greatly supplemented by coordination between various chromium(III) nods and glutamate carboxylates, complemented by numerous hydrogen bonds among the folates, solvent molecules (DMSO, water), and acetates (Figure 11, Scheme S1).

We show that the FA‐Cr(III)‐acetate gel is a potential candidate for oil recovery applications based on our proof‐of‐concept experiments as it overcomes the currently known limitations of various polyacrylamide‐based polymeric gels. The folic acid gelator and the FA‐Cr(III)‐acetate‐based gels in particular show multiple beneficial features in a single system, which were not found together in any other gelation system for EOR. Results of the laboratory study suggest that this novel gel technology should be operational and environmentally attractive for field tests. The non‐toxic and natural FA is a broad spectral gelator with various metal salts, forming saline‐ and thermo‐resistant adjustable gels. The FA can be a gelator for high and low permeability zones depending on gel formulation and application. The gels introduced in our technology represent a single‐fluid system, i.e., the simple injection of a single concentrated DMSO solution to a seawater phase in situ leads to gelation, where the strength of the gel (ranging from highly flowing to rigid, rubbery gels) can be controlled by the choice of metal salt or by varying the concentrations or the component ratio, which ultimately makes them applicable to a wide range of oilfield requirements and environmental and geological conditions. As stable gels can be achieved under various conditions and over a substantial range of FA to metal salts ratios, suggesting that precise formulations, which might be generally difficult to achieve during fieldwork, are not necessary prerequisites for practical and successful applications of the gels’.

The chromium(III) acetate gels show beneficial thermal properties, high stability (up to 200°C), and slow formation rate at 150°C which provides a reasonable delay of gelation necessary for thorough penetration of the gelating mixture in the reservoir matrix. The gels show undisturbed (or even elevated) ability of gelation in seawater (also at an elevated temperature of 150°C) and long‐term stability in (sea)water or a highly saline saturated environment. Moreover, their thixotropic behavior could provide benefits in certain oil mining applications where injection of preformed gel is desirable, i.e., the gel technology can also be used in high permeability zones, and the quality of the gel does not change by shearing during pumping or flowing through pore throats, being it also capable to perform under dynamic reservoir conditions.

DMSO as a co‐solvent does not pose any significant environmental risk^[^ ^73^, ^74^, ^75^ ^]^ and can have some additional benefits, e.g., it helps to improve the thermal stability of gels, decreases the total volume needed for in situ injection, and shows the higher viscosity and good miscibility with an aqueous solution that is related to the slow diffusion throughout the aqueous volume that supports the formation of a homogenous and stable gel.

Finally, this study shows FA in combination with various metal salts to be a powerful gelator and predestines it to become an interesting, biocompatible, and sustainable building block for the materials chemistry of the future. We believe that because of their biocompatibility, these materials could also find uses in other laboratory and industrial processes, especially in the pharmaceutical and biomedical sciences and technologies.

## Conflict of Interests

M.A.M., R.M., and O.J. have filed a patent application regarding this work. The authors declare no other competing interests.

## Supporting information



Supporting Information

Supplemental Video 1

Supplemental Video 2

## Data Availability

The data that support the findings of this study are available from the corresponding author upon reasonable request.
