# Supplementary material for: Robust Self‐Healing Metallo‐Supergels of Folic Acid: Potential Sustainable Gelator for Oilfield Applications
Source: Chemistry. 2025 Apr 9;31(25):e202500748. doi: 10.1002/chem.202500748 (PMC12057599; doi:10.1002/chem.202500748)
Supplement: Supplementary file 1 — Supporting Information [file CHEM-31-e202500748-s002.pdf]

Supporting Information
©Wiley-VCH 2021
69451 Weinheim, Germany

Robust Self-Healing Metallo-Supergels of Folic Acid: Potential Sustainable Gelator for Oilfield Applications

Mahya Asgharian Marzabad, Subhasis Chattopadhyay, Sami Hietala, Nonappa,* Radek Marek, Ondřej Jurček*

Abstract: Majority of known metallogels are based on carefully designed ligands using extensive chemical synthesis and their gelation is often limited to a specific metal salt only. Here, we demonstrate that natural and readily available folic acid (FA) can act as a supergelator in presence of a wide group of metal salts. In this systematic investigation we have prepared and characterized 17 mechanically robust FA-metallogels (0.2wt% or less). Using oscillatory rheological measurements, we further show that the metallogels undergo rapid recovery and self-healing, recovering up to 95% of their original stiffness within a minute. Among studied metallogels, FA-chromium(III) acetate gel (0.4wt%) displayed the highest stiffness with a storage modulus of 4 kPa. We further indicate that the stiffness, recovery, and sol ↔ gel transitions can be readily tuned by either changing metal salt or concentration. Using a combination of various analytical methods we also suggest a structure of self-assembly in the metallogel network. This study defines the biologically important FA as a powerful and sustainable building block of metallogels – mechanically tunable, multi-responsive soft materials. Moreover, performing a proof-of-concept experiments we demonstrate that the FA-chromium(III) gel can be considered as a potent sustainable gellator for enhanced oil recovery applications.

Table of Contents

Experimental Procedures....4
Materials.... 4
Preparation of FA Metallogels.... 4
Characterization.... 4
Rheology.... 4
Fourier Transformed Infrared Spectroscopy (FT-IR).... 5
Circular Dichroism (CD).... 5
UV-Vis Spectroscopy.... 5
^{1}H-NMR and Variable Temperature (VT) ^{1}H-NMR Spectroscopy.... 5
Powder X-Ray Diffraction (PXRD).... 5
Differential Scanning Calorimetry (DSC).... 5
Scanning Electron Microscopy (SEM).... 6
Transmission Electron Microscopy (TEM).... 6
Results and Discussion.... 6
Figure S1.... 6
Figure S2.... 6
Table S1.... 7
Figure S3.... 7
Figure S4.... 8
Table S2.... 9
Figure S5.... 9
Table S3.... 10
Figure S6.... 10
Table S4.... 11
Figure S7.... 11
Table S5.... 12
Figure S8.... 13
Figure S9.... 14
Figure S10.... 15
Scheme S1.... 16
Figure S11.... 17
Figure S12.... 17
Figure S13.... 18
Figure S14.... 18
Figure S15.... 19
Figure S16.... 19
Figure S17.... 20
Figure S18.... 20
Figure S19.... 21

Figure S20 22
Figure S21 23
Table S6 23
Figure S22 25
Table S7 25
Table S8 26
Figure S23 26
References 26
Author Contributions 26

Experimental Procedures

Materials

Folic acid (FA) (>98.0%) was purchased from TCI (Tokyo Chemical Industry). Sodium acetate trihydrate, sodium nitrate, sodium chloride, magnesium(II) acetate (99%), magnesium(II) nitrate hexahydrate (98%), magnesium(II) chloride hexahydrate (98%), potassium acetate trihydrate, potassium nitrate, potassium chloride, calcium(II) acetate hydrate, calcium(II) nitrate tetrahydrate, calcium(II) chloride, chromium(III) acetate basic, chromium(III) nitrate nonahydrate (99%), chromium(III) chloride hexahydrate (99%), manganese(II) acetate tetrahydrate (99%), manganese(II) nitrate tetrahydrate (98%), iron(II) acetate, iron(III) nitrate nonahydrate, iron(III) chloride hexahydrate, cobalt(II) acetate tetrahydrate (98%), cobalt(II) nitrate hexahydrate (99%), cobalt(II) chloride hexahydrate (98%), nickel(II) acetate tetrahydrate (98%), nickel(II) nitrate hexahydrate (99.9%), nickel(II) chloride hexahydrate (98%), copper(II) acetate monohydrate (98%), copper(II) nitrate tetrahydrate (99.5%), copper(II) chloride dihydrate (99%), zinc(II) acetate dihydrate, zinc(II) nitrate hexahydrate and zinc(II) chloride salts were purchased from Abcr GmbH (Germany); manganese(II) chloride tetrahydrate (>98.0%) was purchased from Fluorochem Ltd. Dimethyl sulfoxide (DMSO) was purchased from Lach-Ner. The simulated seawater was prepared by dissolving NaCl (0.48 M), $MgSO_4$ (0.03 M), $CaCl_2$ (0.01 M), and KCl (0.01 M) in distilled water (salinity 33.5 g/L). The brine solution (saturated) was prepared dissolving NaCl (4.8 M), $MgSO_4$ (0.3 M), $CaCl_2$ (0.1 M), and KCl (0.1 M) in distilled water (salinity 335 g/L).

Preparation of FA Metallogels

General procedure of 0.4 wt% metallogel preparation is as follows, 4.0 mg of FA powder was added into a vial containing 0.5 mL of DMSO (DMSO-d_6 for ^{1}H NMR analysis) and equipped with a magnetic stir bar. The mixture was heated to 70 °C under stirring. Meanwhile, 1 equimolar amount of metal salt was dissolved in 0.5 mL of distilled water (D_2O for ^{1}H NMR analysis). After complete dissolution of both components, the solution of metal salt was added to the FA solution. The resulting mixture was stirred at 70 °C until a transparent solution was formed. The final solution was cooled down to room temperature and kept at that temperature without any disturbance. The gelation was recognized by inverting the glass vial (Fig. 1 and Fig. S2) - gelation time varied from minutes to days depending on the metal salts used. All metallogels were prepared using the same approach (Table S1) where the weight of FA and metal salts were also modified based on the desired concentration of the gel.

Characterization

Rheology

Because of the gel preparation procedure for some of the samples, the *in situ* gelation in the rheometer was not possible. Therefore, premade and stabilized gels were used for all the experiments. For all experiments, TA AR2000 stress-controlled rheometer with parallel-plate geometry (20 mm steel plate) in constant distance of 1.6 mm, equipped with a Peltier heated plate was used. The measuring setup was covered with a sealing lid to prevent solvent evaporation. First oscillatory amplitude sweep studies were conducted from 0.01 to 200% strain with an angular frequency of 6.28 rad/s at 20 °C to establish the linear viscoelastic region (LVE) of the gels. Time-sweep studies up to 60 min and oscillatory frequency sweeps were performed after the loading using a strain within the LVE range (0.1%). For step-strain experiments, the gels were subjected for alternating 0.1% and 150% strains for 60 s for each cycle. Temperature sweep experiments were carried out in temperature ramps from 20 to 90 °C (heating cycle) and from 90 to 20 °C (cooling cycle) with 0.1% strain amplitude and 5 °C/min heating rate. For all the samples, experiments were carried out in duplicates.

Fourier Transformed Infrared Spectroscopy (FT-IR)

FT-IR spectra were measured at room temperature with a Nicolet 6700 FT-IR spectrometer (USA) equipped with a standard mid-IR source, KBr beam splitter, and DTGS detector and with the cell compartment purged by dry nitrogen during all the measurements. The FT-IR spectra were obtained by placing a known amount of the gel samples directly on sample slot. The spectra were collected at the spectral range 600–4000 cm^{-1}, with a resolution 2 cm^{-1}, 512 scans, using Happ-Genzel apodization function.

Circular Dichroism (CD)

A FA solution (0.2 wt%) in DMSO (40 µL) and a FA (0.2 wt%) with chromium(III) acetate in DMSO (40 µL) were measured first. Then a FA gel and a FA-Cr(III)-acetate metallogel in DMSO:H2O mixture 1:1 were measured for comparison (Fig. 6). The gel samples were prepared, thus: FA solution (0.2 wt%) in DMSO (20 µL) was placed in a cuvette, water (20 µL) was added, and the resulting solution was mixed. For the metallogel, an aqueous solution (20 µL) of chromium(III) acetate (FA:metal 1:1) was added to the FA solution (0.2 wt%) in DMSO (20 µL) and mixed. The CD spectra of DMSO and DMSO:water were measured as blank. CD spectra were recorded using spectropolarimeter Jasco J-815. The spectra were recorded at 25 °C in 0.2 mm cell in the range 450 - 250 nm (data pitch 1 nm, D.I.T. 2 sec, bandwidth 1.00 nm, scanning speed 100 nm/min, and 6 accumulations).

UV-Vis Spectroscopy

UV-visible absorption spectra were recorded using Agilent Cary 60 spectrophotometer (USA) with customized sample chamber (which can be fitted with cuvettes and solid samples) in the range of 200-800 nm. The UV-Vis spectra of neat FA in DMSO, FA in DMSO:water, and FA-metallogel (all at 0.4 wt%) were recorded at room temperature with 1 mm quartz cuvette or fitting the solid gel samples in the holder. The position and shift of the absorption peaks were used to analyze the interaction between FA molecules and metal salts.

^{1}H-NMR and Variable Temperature (VT) ^{1}H-NMR Spectroscopy

^{1}H NMR spectra were recorded using a Bruker Avance III HD 700 MHz spectrometers. All the ^{1}H NMR spectra are referenced to the residual internal signal of DMSO-d_5 of DMSO-d_6 solvent (2.5 ppm). The temperature was gradually changed from 298.2 K to 348.2 K for variable temperature ^{1}H NMR experiments. Data were analysed using Mestrenova Program (v1.13).

Powder X-Ray Diffraction (PXRD)

The crystallinity and phase purity of synthesized gel samples were determined by PXRD experiments after freeze drying. The samples were pressed on an aluminium slide, sample height was aligned with laser, and data were collected in the desired range (3°<2θ<90°). The diffraction data were collected on a RIGAKU SmartLab 3 kW diffractometer with a fine focus Cu sealed tube with graphite monochromated MoKα at 40 kV, 30 mA.

Differential Scanning Calorimetry (DSC)

Thermogravimetric data of the samples were acquired using Perkin Elmer STA 6000 thermogravimetric TG/DSC analyser run under Pyris™ software. DSC curves were recorded under air atmosphere (flow rate of 40 mL/min) with a heating rate of 10 °C/min and temperature range of 20-95 °C for the wet gel samples and 22-622 °C for dry gel samples. The wet samples were prepared in an aluminium cup with a hermetic lid, the dry samples were measured in an open platinum holder. The sample weights were varying from 4 to 10 mg. Temperature calibration of the analyser was made using melting points of the indium (156.6 °C), zinc (419.5 °C), and aluminium (660.3 °C) standards. The weight balance was calibrated with standard weight at room temperature.

Scanning Electron Microscopy (SEM)

For SEM imaging, the preformed metallogels were freeze-dried. The resulting aerogels were placed onto a conductive carbon tape placed on an aluminium stub. The specimen was sputter coated with gold. For SEM imaging, field-emission scanning electron microscope (FEI Versa 3D SEM or Carl Zeiss AG – EVO®50 Series SEM) was used.

Transmission Electron Microscopy (TEM)

For TEM, specimen preparation was carried out by adding 3-5 µL of the sample on a 300-mesh copper grid with holey carbon support film. In all cases premade gel was heated until it turned into a clear solution and the hot solution was used for drop casting. The excess liquid was blotted using filter paper before drying under ambient conditions. The images were acquired using Jeol F200 S/TEM microscope operated at 200 keV and Gatan Digital Micrograph® software.

Results and Discussion

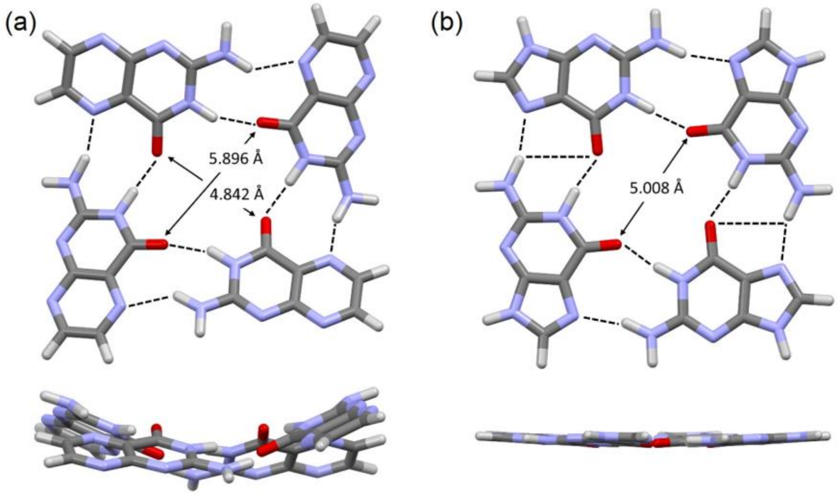

Figure S1. Models of (a) pterin (Ptr) quadruplex (E_h = -2321.97 Hartrees) and (b) guanine (G) quadruplex (E_h = -2169.65 Hartrees). Dashed lines represent hydrogen bonds. The size of the void inside quadruplexes is described by intermolecular distance between oxygen atoms of opposite lying molecules of heterocycle. While the Ptr-quadruplex shows lower symmetry and slight out of plane organization with diameters 5.896 Å and 4.842 Å, the G symmetric and planar tetrad possesses 5.008 Å diameter, suggesting that the void of Ptr-quadruplex could be slightly larger possibly having the ability to accommodate larger cations than those usual for G-quadruplex (K or Na). Interestingly, previous more detailed studies suggest that fit of Na and K towards G- and Ptr-complexes goes in reverse, while G-quadruplex shows higher affinity towards K, the Ptr-quadruplex prefers Na.[1,2,3] The models were optimized using DFT calculation with ωB97X-D method and 6-31G* basis set.

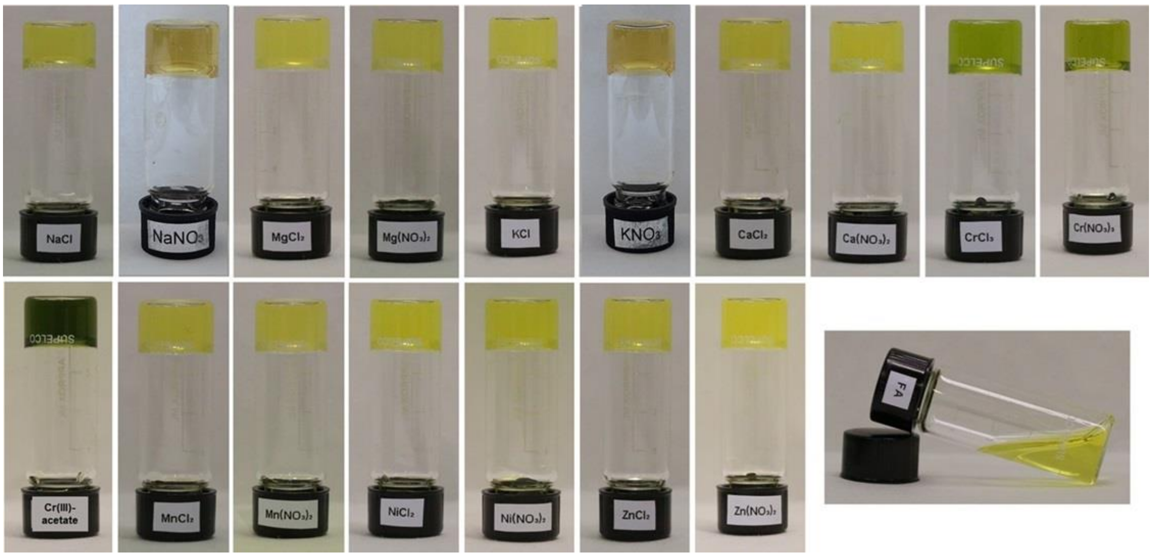

Figure S2. Folic acid-metal salts gel formation in DMSO:water mixture (1:1) at 0.2 wt%.

Table S1. Folic acid-metal salts gel formation in DMSO:water mixture (1:1) at 0.4 % (all % refers to w/v% unless stated otherwise). S refers to solution, P to precipitate, and G refers to gel formation.

Metal Salt	Amount	Result	Metal Salt	Amount	Result	Metal Salt	Amount	Result
NaAcO	1.2 mg	S	NaCl	0.6 mg	G	$NaNO_3$	0.8 mg	G
$Mg(AcO)_2$	1.3 mg	P	$MgCl_2$	1.8 mg	G	$Mg(NO_3)_2$	2.3 mg	G
KAcO	1.3 mg	S	KCl	0.7 mg	G	KNO_3	0.9 mg	G
$Ca(AcO)_2$	1.4 mg	S	$CaCl_2$	1.0 mg	G	$Ca(NO_3)_2$	2.1 mg	G
Cr(III)-acetate	5.4 mg*	G	$CrCl_3$	2.4 mg	G	$Cr(NO_3)_3$	3.6 mg	G
$Mn(AcO)_2$	2.2 mg	S	$MnCl_2$	1.8 mg	G	$Mn(NO_3)_2$	2.3 mg	G
$Fe(AcO)_2$	1.6 mg	P	$FeCl_3$	2.4 mg	P	$Fe(NO_3)_3$	3.6 mg	P
$Co(AcO)_2$	2.2 mg	S	$CoCl_2$	2.1 mg	S	$Co(NO_3)_2$	2.6 mg	S
$Ni(AcO)_2$	2.2 mg	S	$NiCl_2$	2.1 mg	G	$Ni(NO_3)_2$	2.6 mg	G
$Cu(AcO)_2$	1.8 mg	S	$CuCl_2$	1.5 mg	P	$Cu(NO_3)_2$	2.2 mg	S
$Zn(AcO)_2$	2.0 mg	S	$ZnCl_2$	1.2 mg	G	$Zn(NO_3)_2$	2.6 mg	G

* Chromium(III) acetate basic (M_W = 603.32 g/mol) is a trimeric cluster, $[Cr_3O(O_2CCH_3)_6(OH_2)_3](O_2CCH_3)$, with a well-defined and stable Cr_3O core stabilized by acetate ligands and a central μ_3-oxo bridge. The amount is calculated to *tris*-chromium(III) cluster as one equivalent to FA. Thus, one molar equivalent of the basic chromium(III) acetate corresponds to three molar equivalents of chromium atoms (FA:Cr(III)-acetate ratio 1:1 corresponds to 1:3 ratio of FA:Cr(III)).

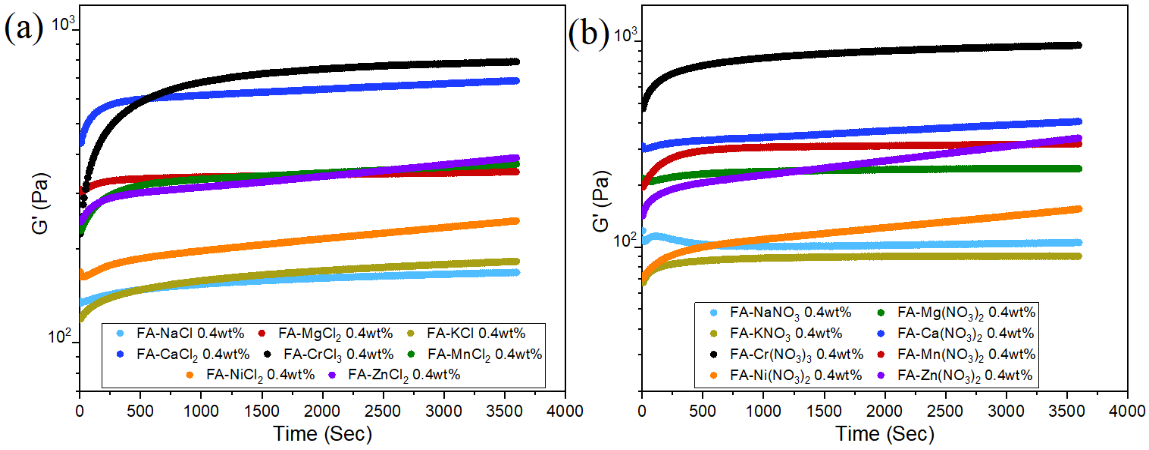

Figure S3. Time-sweep experiments for 0.4% DMSO:water (1:1) gels (a) comparing chloride of all cations, (b) comparing nitrate of all cations.

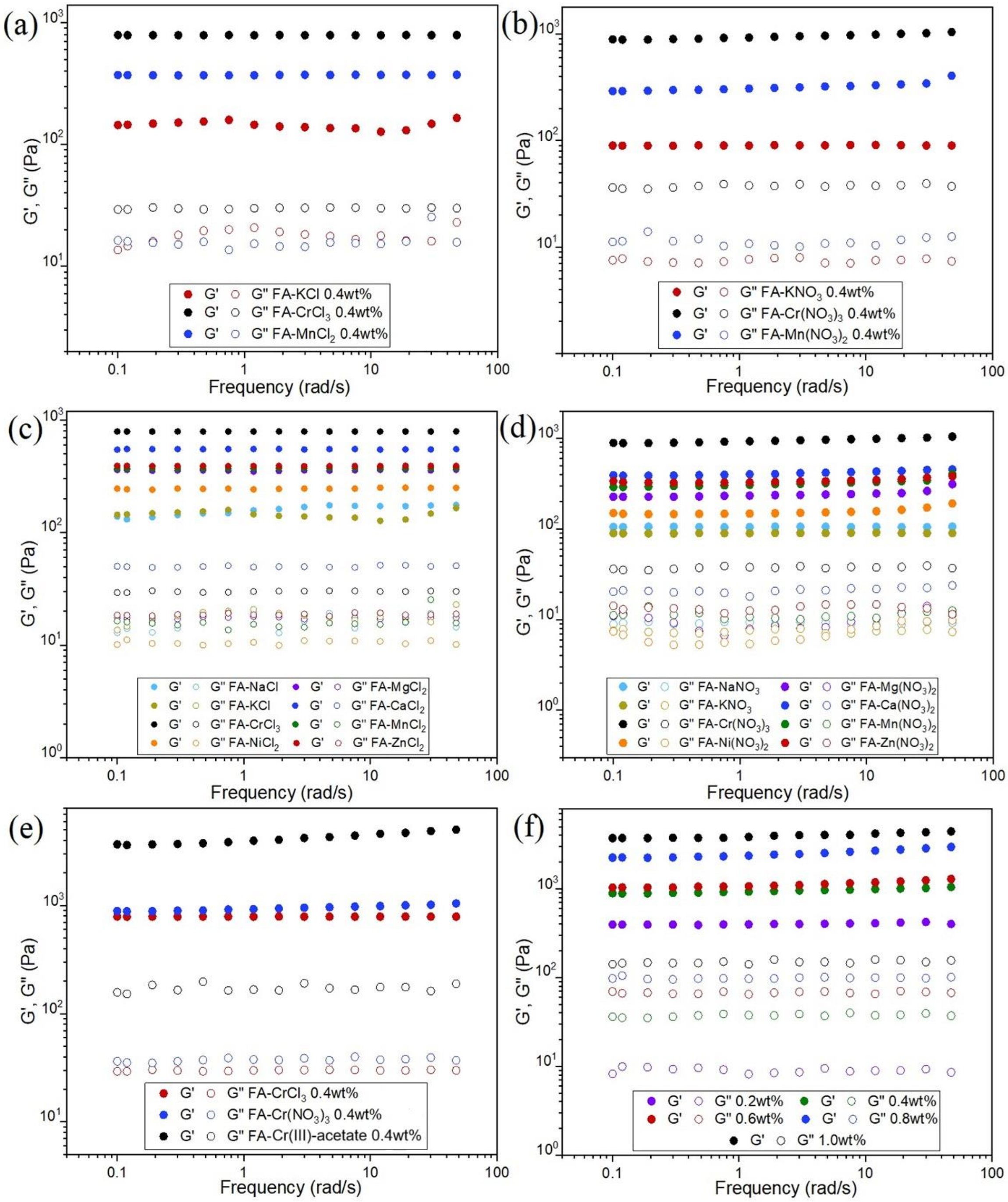

Figure S4. Frequency-sweep experiments for 0.4 % DMSO:water (1:1) gels (a) comparing chlorides of mono- di- and trivalent cations, (b) comparing nitrates of mono- di- and trivalent cations, (c) comparing chloride of all cations, (d) comparing nitrate of all cations, (e) comparing the effect of anion in Cr(III) salt, and (f) study the effect of FA concentration in $Cr(NO_3)_3$ gel samples.

Table S2. G′ and G″ of metallogels (in DMSO:H_2O 1:1 by volume, 0.4 % FA, 1 mL total volume) measured in frequency-sweep experiments, shown at 0.1 rad/s. FA = folic acid.

Metal salt	G′ (Pa)	G″ (Pa)
NaCl	138.9	12.9
$MgCl_2$	360.2	17.56
KCl	144.1	13.63
$CaCl_2$	545.3	50.06
$CrCl_3$	790.5	29.3
$MnCl_2$	371.8	16.36
$NiCl_2$	245.8	10.12
$ZnCl_2$	390.5	18.51
$NaNO_3$	105.3	8.89
$Mg(NO_3)_2$	227.2	10.96
KNO_3	89.86	7.504
$Ca(NO_3)_2$	391.7	20.34
$Cr(NO_3)_3$	891	36.25
$Mn(NO_3)_2$	290.8	11.17
$Ni(NO_3)_2$	149.7	7.408
$Zn(NO_3)_2$	337.7	14.22
Cr(III)-acetate	3685	157.8

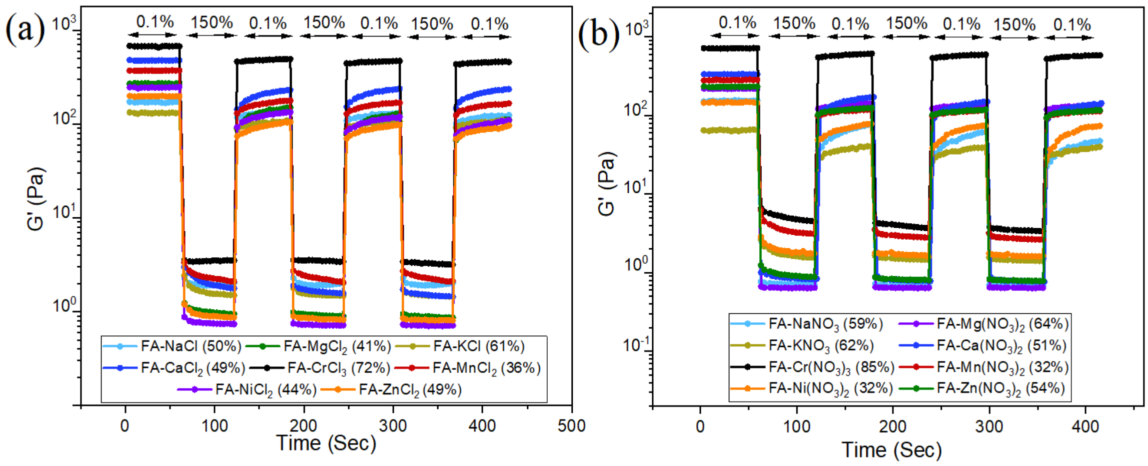

Figure S5. Step strain experiments for 0.4 wt% DMSO:water (1:1) gels (a) comparing chloride of all cations, (b) comparing nitrate of all cations.

Table S3. G′ at frequency strain 0.1 rad/s and its recovery after three gel ↔ sol cycles (FA 0.4 %, metal salt:FA = 1:1 (molar), in DMSO:H_2O 1:1 by volume).

Metal salt	G′ (Pa)	Recovery (%)
NaCl	138.9	50
$MgCl_2$	360.2	41
KCl	144.1	61
$CaCl_2$	545.3	49
$CrCl_3$	790.5	72
$MnCl_2$	371.8	36
$NiCl_2$	245.8	44
$ZnCl_2$	390.5	49
$NaNO_3$	105.3	59
$Mg(NO_3)_2$	227.2	64
KNO_3	89.96	62
$Ca(NO_3)_2$	391.7	51
$Cr(NO_3)_3$	891	85
$Mn(NO_3)_2$	290.8	32
$Ni(NO_3)_2$	149.7	32
$Zn(NO_3)_2$	337.7	54
Cr(III)-acetate	3685	95

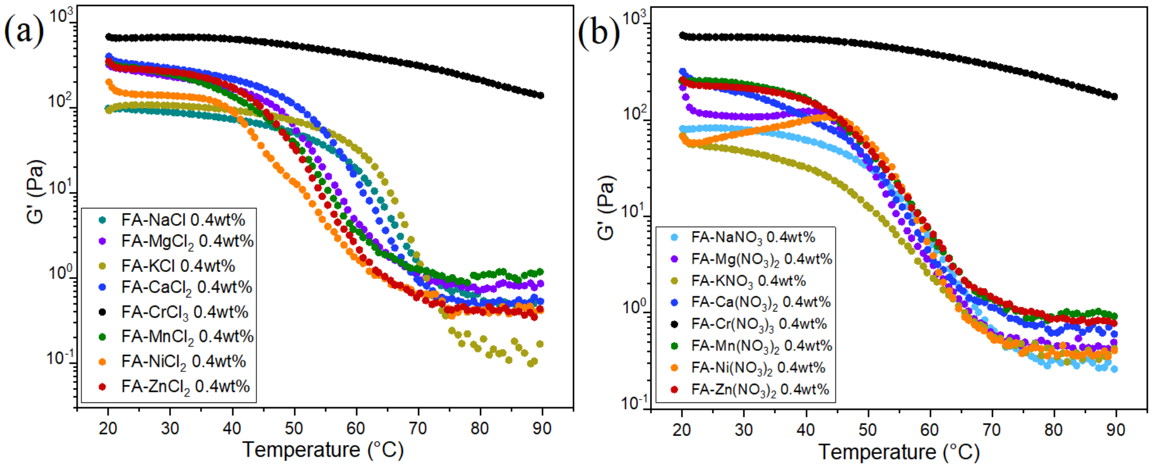

Figure S6. Temperature sweep experiments for 0.4 % DMSO:water (1:1) gels (a) comparing chloride of all cations, (b) comparing nitrate of all cations.

Table S4. Gel melting temperatures (T_{gel}) as screened in temperature-sweep experiments between 20-90 °C (FA 0.4 %, metal salt:FA 1:1 (molar), DMSO:H_2O 1:1 by volume).

Metal salt	Gel melting temperature (T_{gel})
NaCl	65.6
$MgCl_2$	58.6
KCl	68.8
$CaCl_2$	63.7
$CrCl_3$	Does not melt in given range
$MnCl_2$	56.3
$NiCl_2$	55.4
$ZnCl_2$	56.4
$NaNO_3$	62.1
$Mg(NO_3)_2$	56.3
KNO_3	57
$Ca(NO_3)_2$	58
$Cr(NO_3)_3$	Does not melt in given range
$Mn(NO_3)_2$	61.2
$Ni(NO_3)_2$	59.5
$Zn(NO_3)_2$	58.8
Cr(III)-acetate	Does not melt in given range

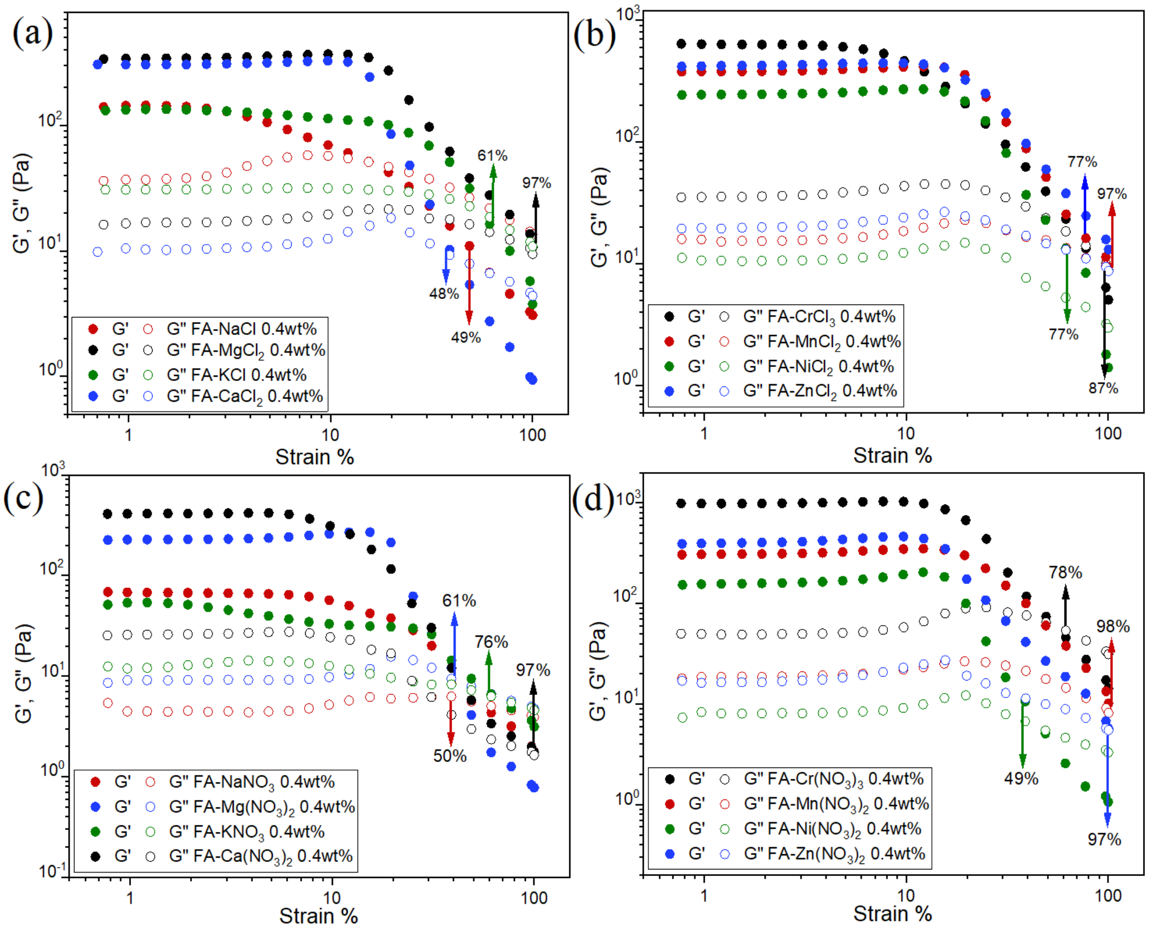

Figure S7. Critical strain experiments for 0.4 % DMSO:water (1:1) gels (a) and (b) comparing chlorides of all cations, (c) and (d) comparing nitrate all cations.

Table S5. Critical strain and yield point of metallogels (FA 0.4 % in DMSO:H_2O 1:1 by volume, metal salt:FA 1:1 (molar)).

Metal salt	Critical strain (%)	Yield point (%)
NaCl	5	49
$MgCl_2$	15.4	97
KCl	24.3	61
$CaCl_2$	15.6	48
$CrCl_3$	12	87
$MnCl_2$	19.5	97
$NiCl_2$	19.5	77
$ZnCl_2$	20	77
$NaNO_3$	19.5	50
$Mg(NO_3)_2$	19.5	61
KNO_3	31	76
$Ca(NO_3)_2$	15.6	97
$Cr(NO_3)_3$	20	78
$Mn(NO_3)_2$	20	98
$Ni(NO_3)_2$	20	49
$Zn(NO_3)_2$	16	97
Cr(III)-acetate	10	49

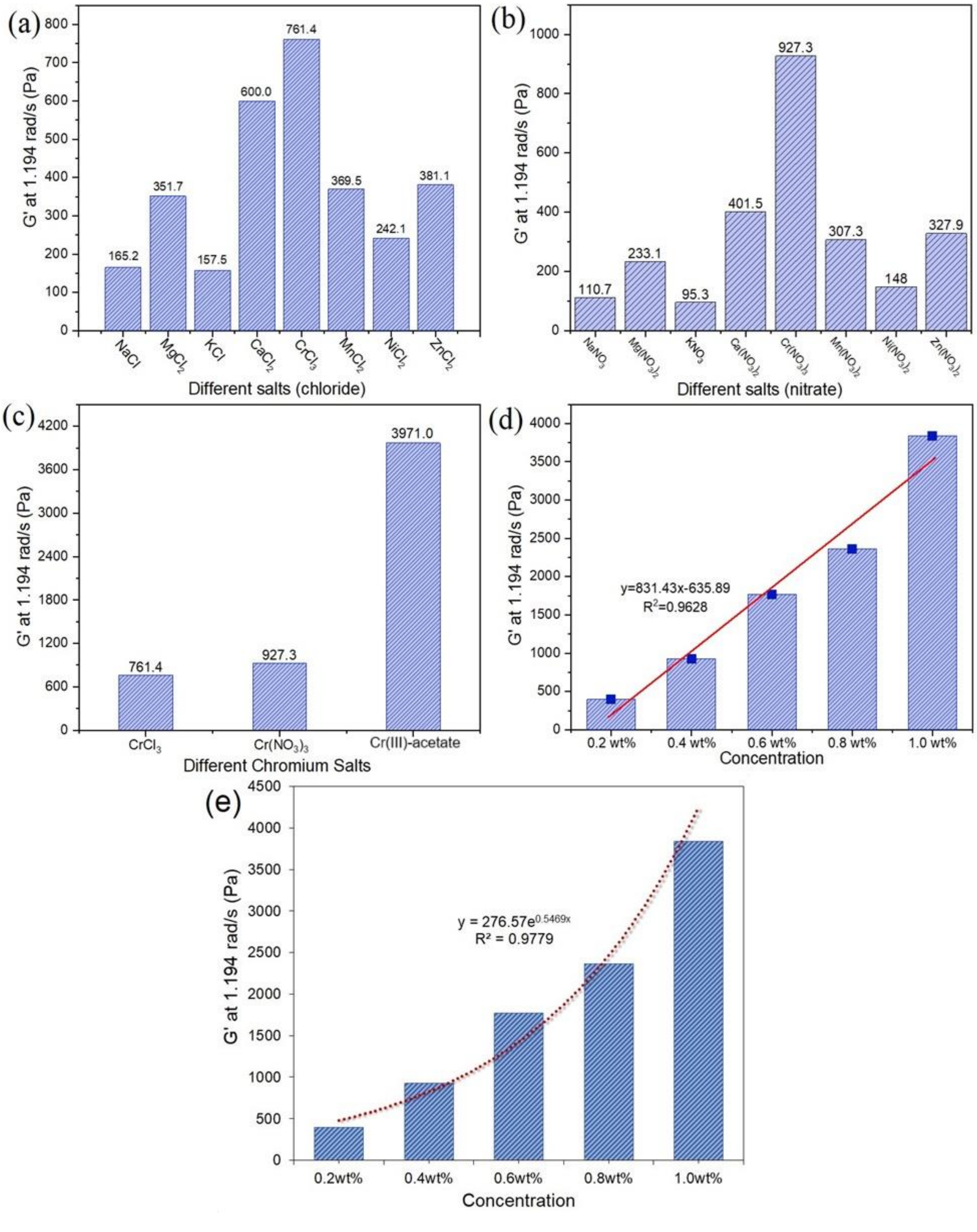

Figure S8. Effect of metal salts and their concentration: (a) comparing different chloride salts, (b) comparing different nitrate salts, (c) comparing the effect of anion on gelation with Cr^{3+}, and study of the effect of $Cr(NO_3)_3$ concentration on G' (d) and (e), linear versus exponential regression, respectively.

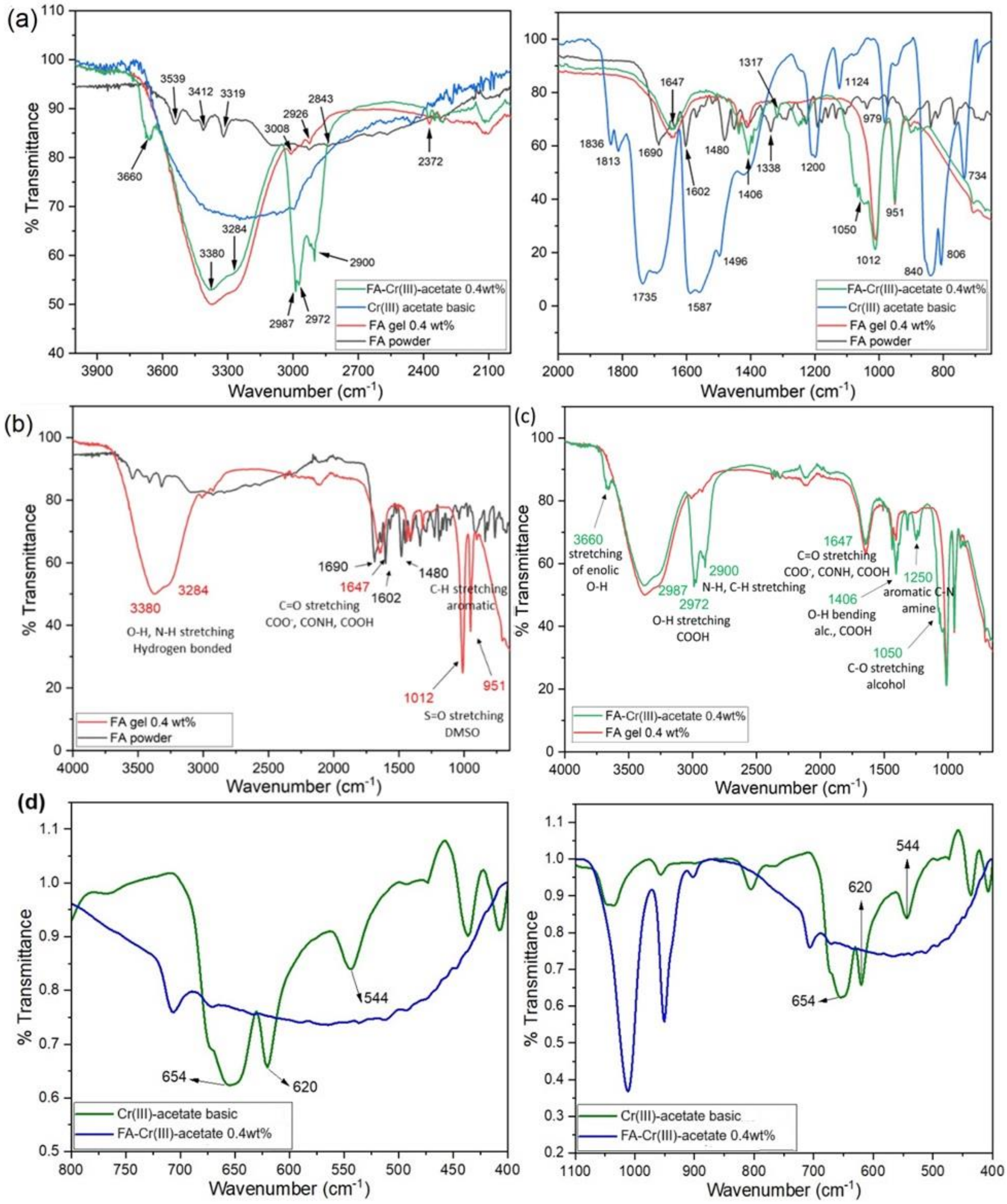

Figure S9. FT-IR spectra of (a) FA powder, Cr(III)-acetate, 0.4 wt% DMSO:water (1:1) FA gel, 0.4 wt% DMSO:water (1:1) FA-Cr(III)-acetate gel, (b) FA powder and 0.4 wt% DMSO:water (1:1) FA gel with highlighted differences, c) 0.4 wt% DMSO:water (1:1) FA gel and 0.4 wt% DMSO:water (1:1) FA-Cr(III)-acetate metallogel with highlighted differences; d) neat chromium(III) acetate basic and FA-Cr(III)-acetate gel (0.4 wt% in DMSO:H_2O 1:1).

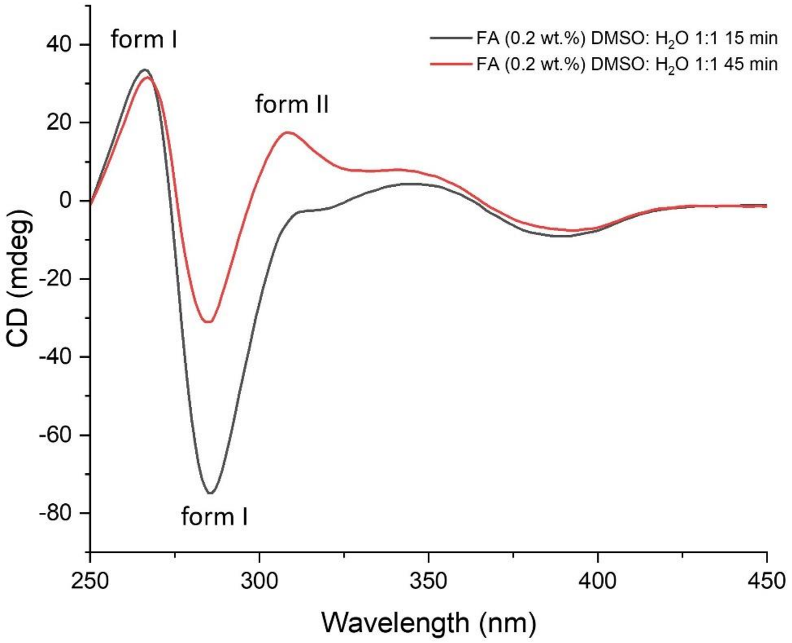

Figure S10. CD spectra of FA-gel: Development of form 2 from a fraction of form 1 in dependance on time, 15 versus 45 min (after this period spectrum did not encounter any additional changes).

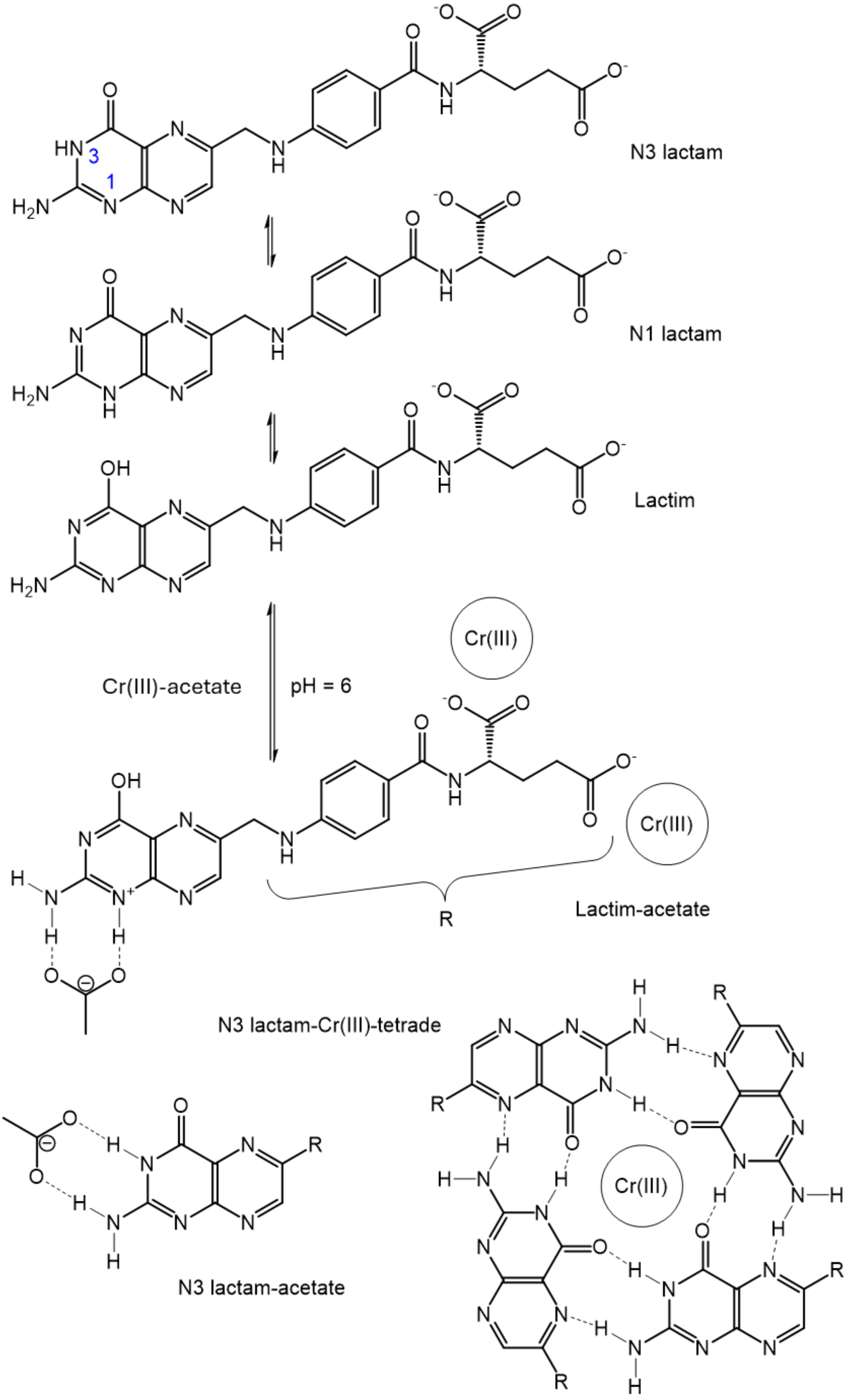

Scheme S1. Keto-enol tautomerization and enol form stabilization of folic acid.

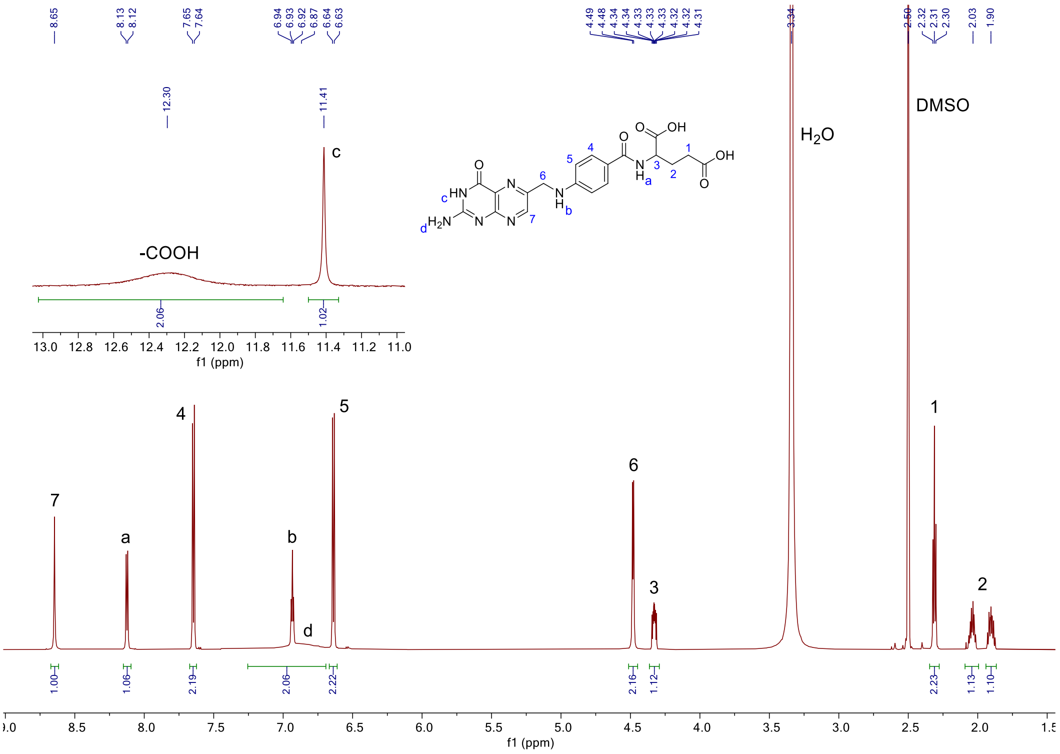

Figure S11. ^{1}H NMR of FA (0.4 wt% in DMSO-d$_6$, 298.2 K, 700 MHz).

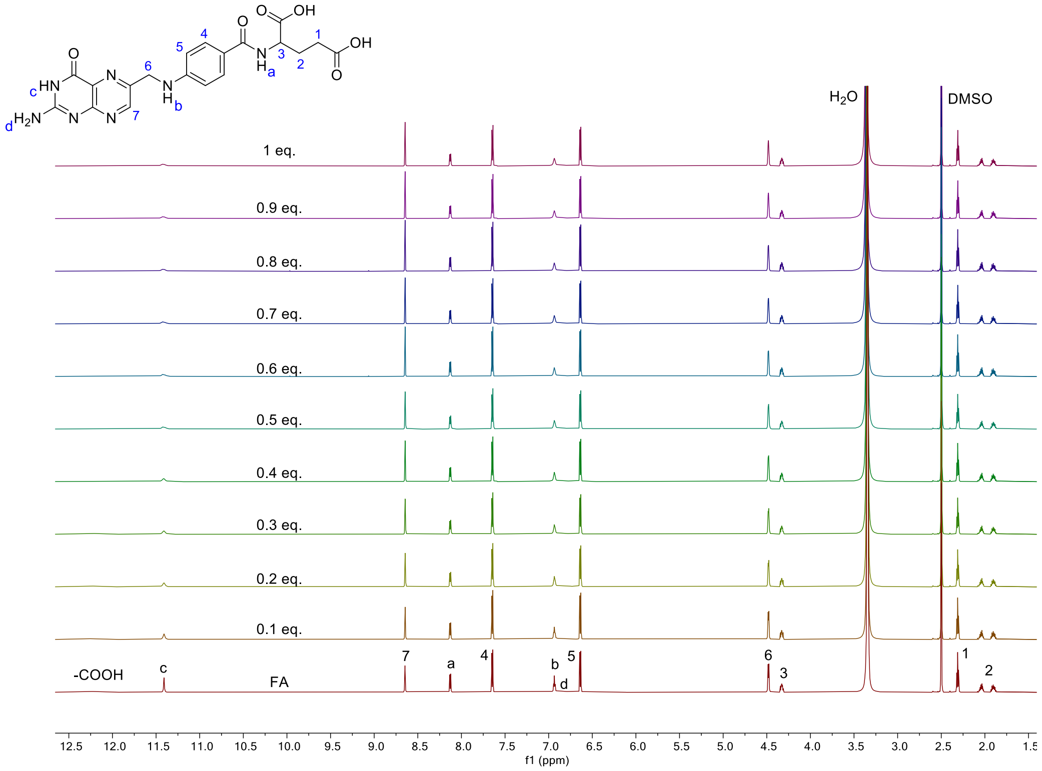

Figure S12. ^{1}H NMR titration study of FA (0.4 wt%) with $Zn(NO_3)_2$ in DMSO-d_6 at 700 MHz and 298.2 K.

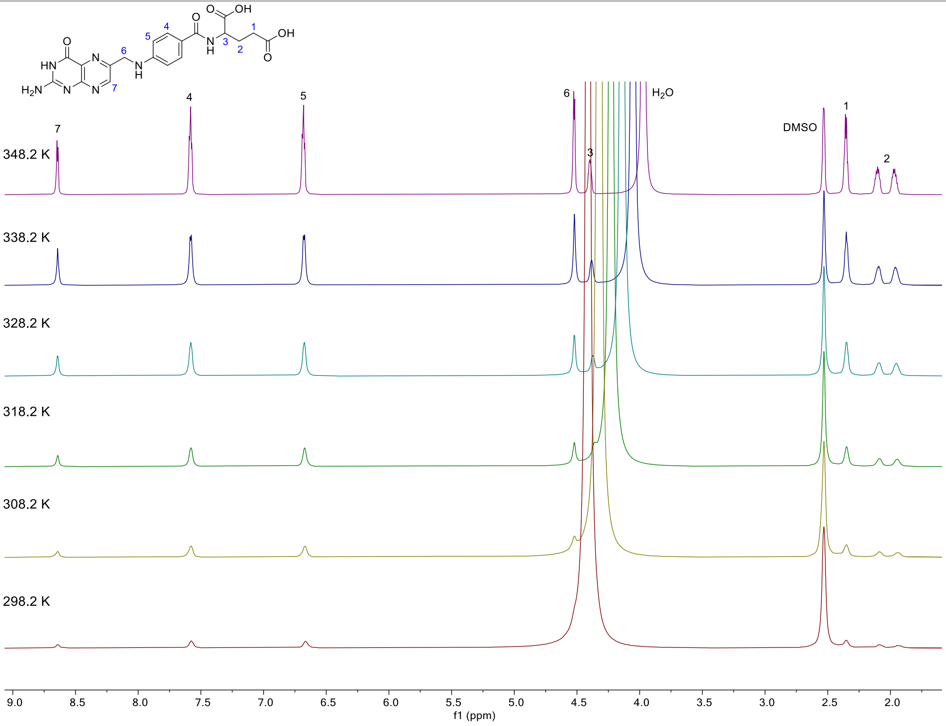

Figure S13. Variable temperature ^{1}H NMR of FA (0.8 wt%) with KCl (M:FA 1:1) in DMSO-d_6:D_2O 1:1 at 700 MHz.

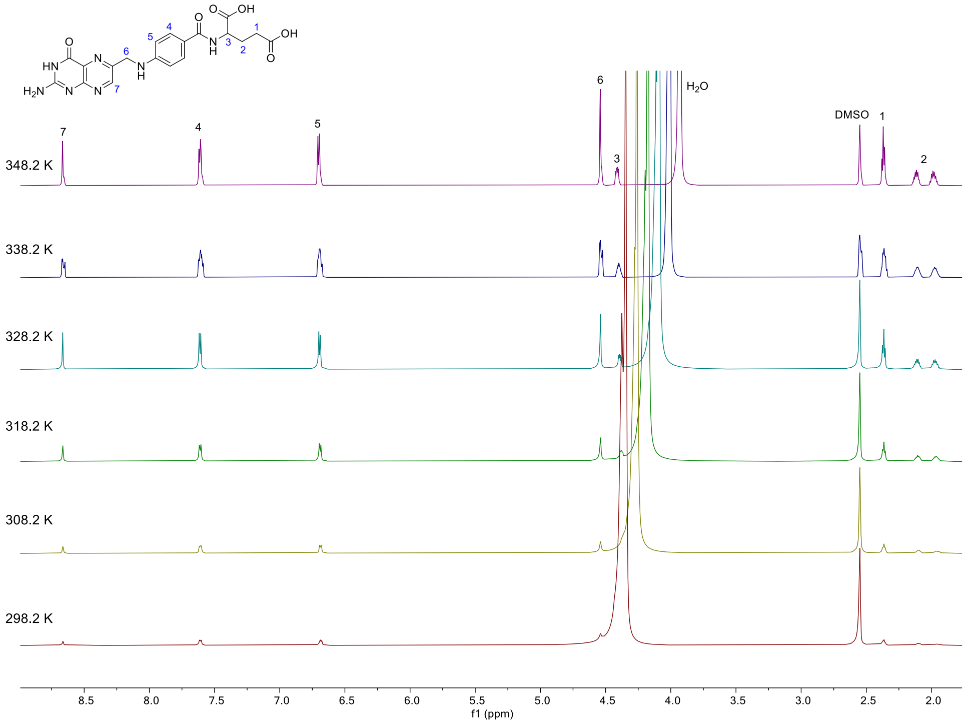

Figure S14. Variable temperature ^{1}H NMR of FA (0.8 wt%) with NaCl (M:FA 1:1) in DMSO-d_6:D_2O 1:1 at 700 MHz.

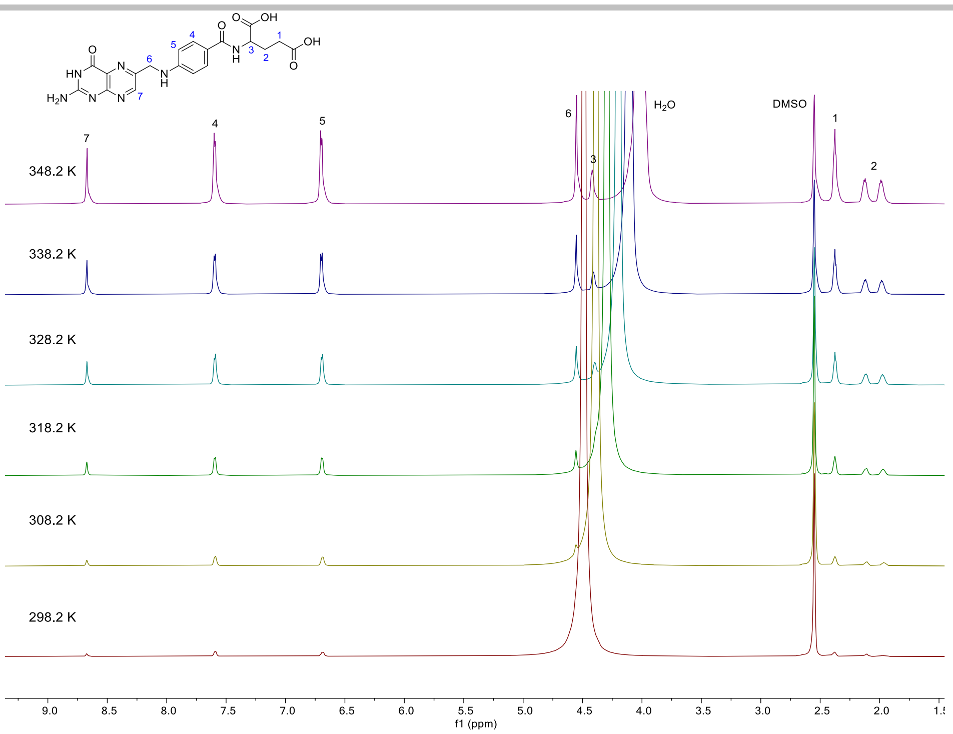

Figure S15. Variable temperature ^{1}H NMR of FA (0.8 wt%) with $Zn(NO_3)_2$ (M:FA 1:1) in DMSO-d_6:D_2O 1:1 at 700 MHz.

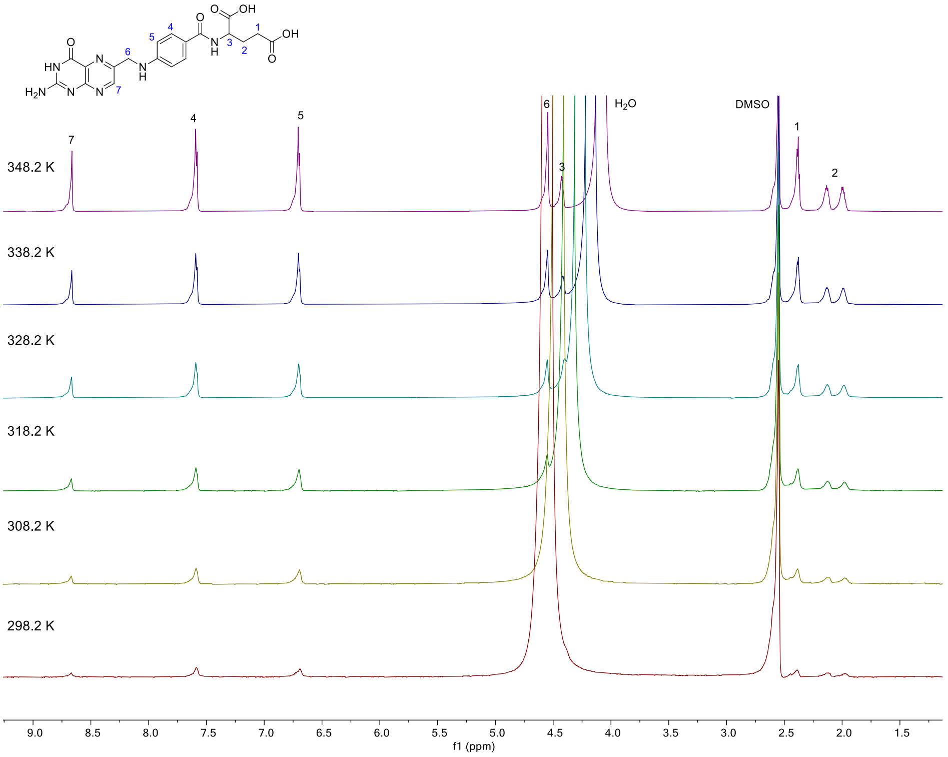

Figure S16. Variable temperature ^{1}H NMR of FA (0.8 wt%) with $ZnCl_2$ (M:FA 1:1) (DMSO-d_6:D_2O 1:1; 700 MHz).

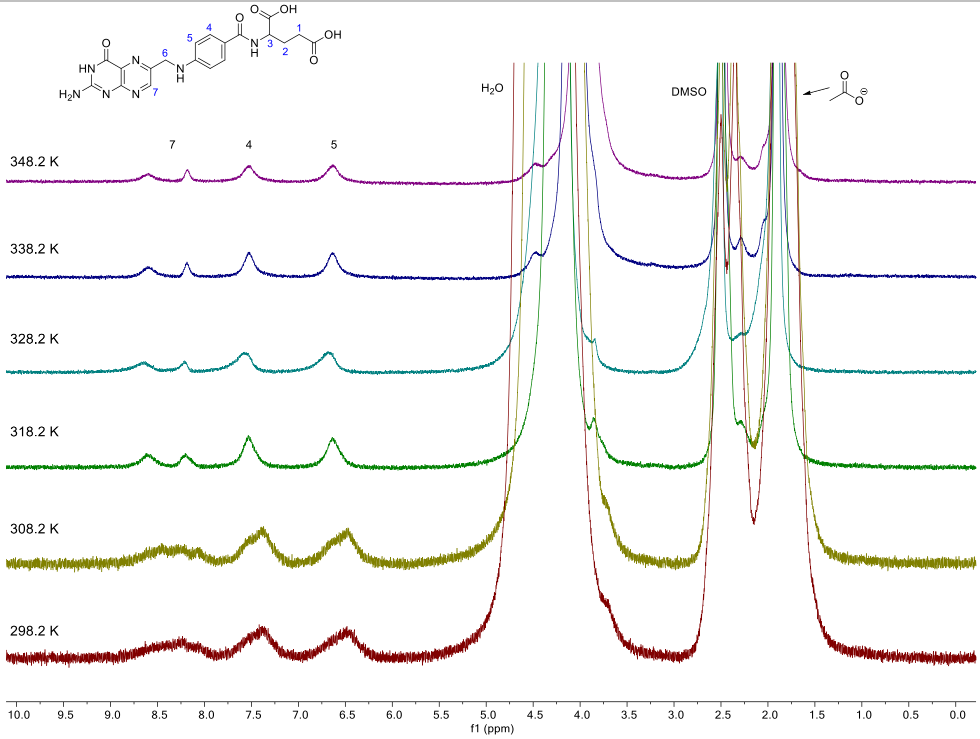

Figure S17. Variable temperature ^{1}H NMR of FA (0.8 wt%) with Cr(III)-acetate (M:FA 1:1) in DMSO-d_6: D_2O 1:1 at 700 MHz.

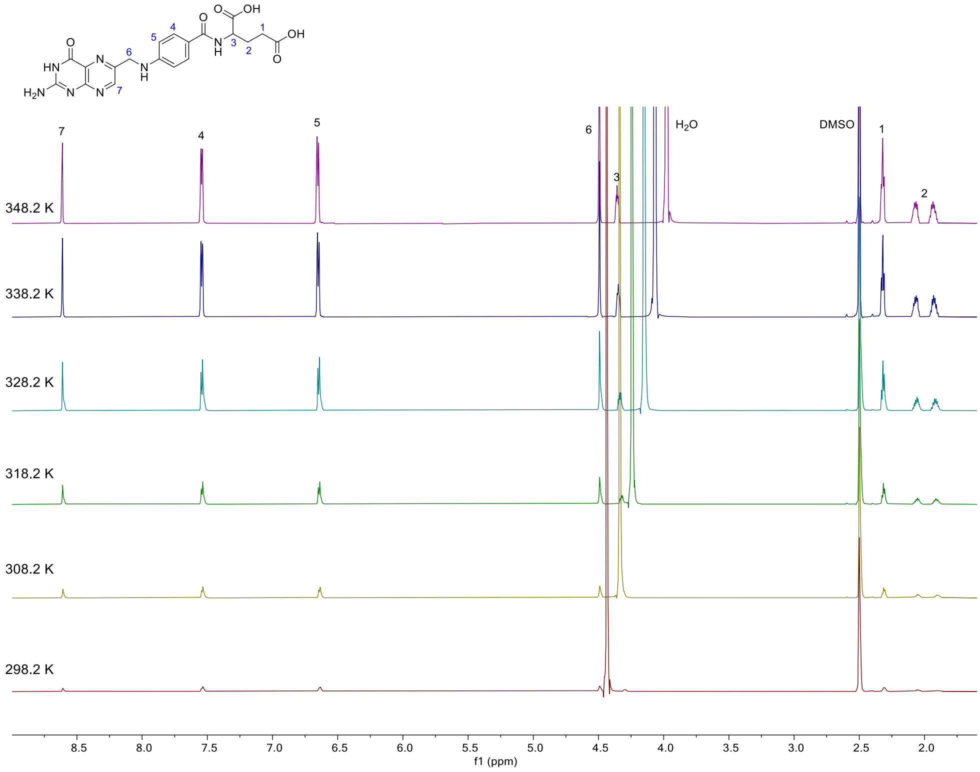

Figure S18. Variable temperature ^{1}H NMR of 0.2 wt% FA gel in DMSO-d_6:D_2O 1:1 at 700 MHz.

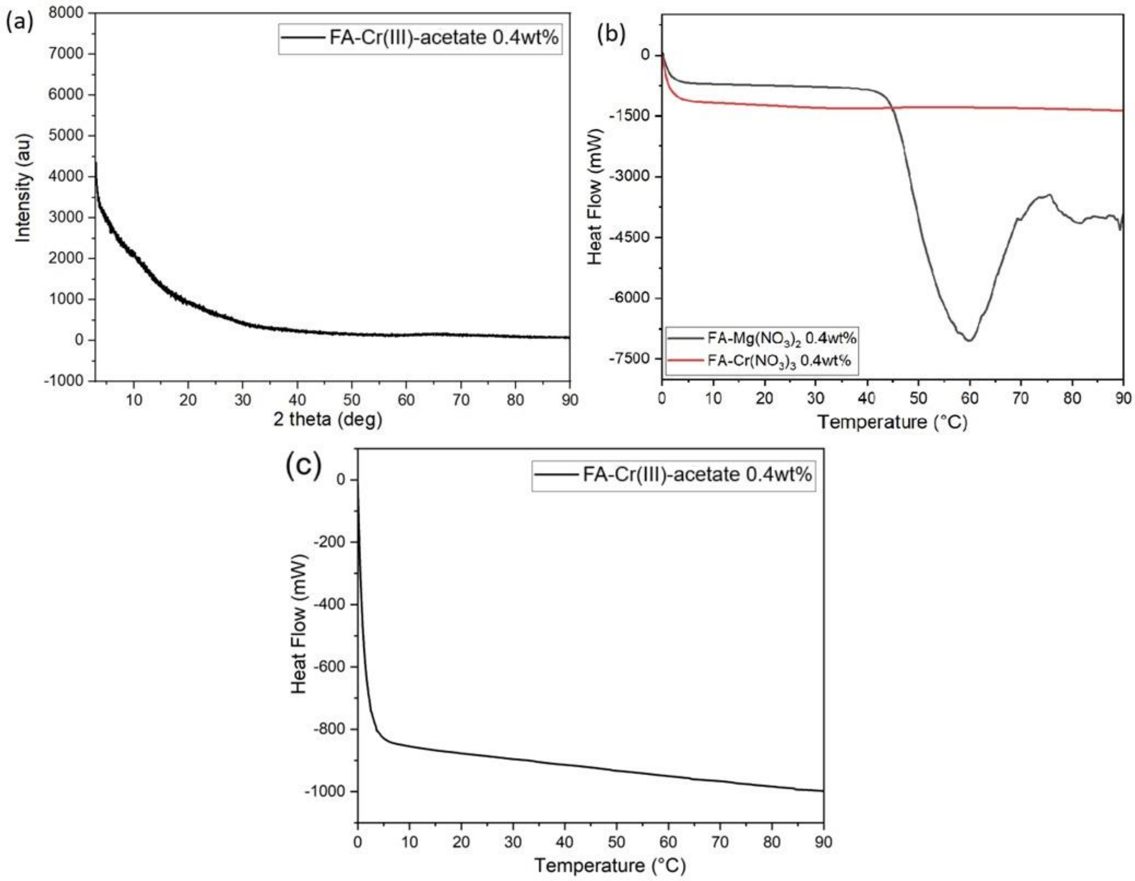

Figure S19. (a) PXRD pattern of freeze dried FA-Cr(III)-acetate 0.4wt% gel sample and (b) DSC curves of FA-$Cr(NO_3)_3$ 0.4wt% and FA-$Mg(NO_3)_2$ 0.4wt% gel samples, (c) DSC curve of FA-Cr(III)-acetate 0.4wt% gel.

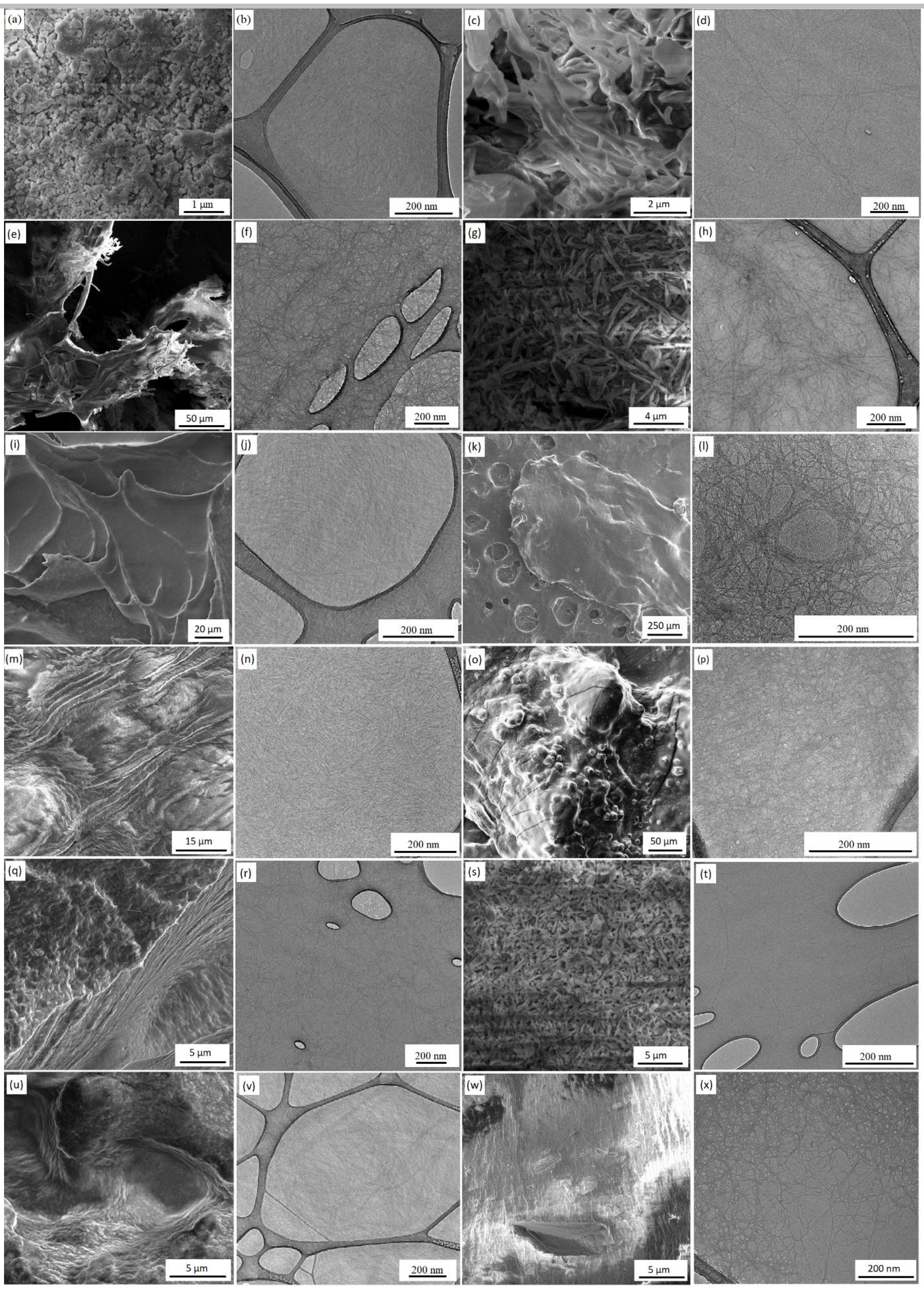

Figure S20. FA-NaCl gel in DMSO:water mixture (1:1) at 0.2 wt% (a) SEM image of freeze dried sample, (b) TEM image. FA-$MgCl_2$ gel in DMSO:water mixture (1:1) at 0.2 wt% (c) SEM image of freeze dried sample, (d) TEM image. FA-$CaCl_2$ gel in DMSO:water mixture (1:1) at 0.2 wt% (e) SEM image of freeze dried sample, (f) TEM image. FA-$NiCl_2$ gel in DMSO:water mixture (1:1) at 0.2 wt% (g) SEM image of freeze dried sample, (h) TEM image. FA-$ZnCl_2$ gel in DMSO:water mixture (1:1) at 0.2 wt% (i) SEM image of freeze dried sample, (j) TEM image. FA-$Mg(NO_3)_2$ gel in DMSO:water mixture (1:1) at 0.2 wt% (k) SEM image of freeze dried sample, (l) TEM image. FA- $Ca(NO_3)_2$ gel in DMSO:water mixture (1:1) at 0.2 wt% (m) SEM image of freeze dried sample, (n) TEM image. FA- $Cr(NO_3)_3$ gel in DMSO:water mixture (1:1) at 0.2 wt% (o) SEM image of freeze dried sample, (p) TEM image. FA- $Mn(NO_3)_2$ gel in DMSO:water mixture (1:1) at 0.2 wt% (q) SEM image of freeze dried sample, (r) TEM image. FA- $Ni(NO_3)_2$ gel in DMSO:water mixture (1:1) at 0.2 wt% (s) SEM image of freeze dried sample, (t) TEM image . FA-$Zn(NO_3)_2$ gel in DMSO:water mixture (1:1) at 0.2 wt% (u) SEM image of freeze dried sample, (v) TEM image. FA- Cr(III)-acetate gel in DMSO:water mixture (1:1) at 0.2 wt% (w) SEM image of freeze dried sample, (x) TEM image.

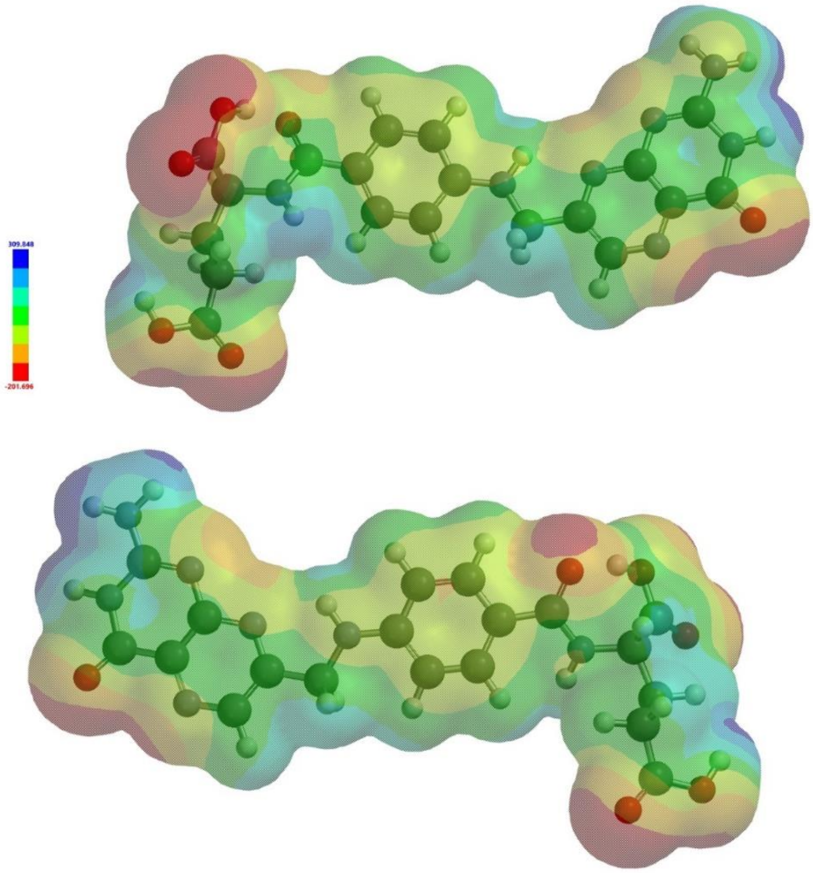

Figure S21. Electrostatic potential map of FA (model of ground state in gas phase using B3LYP/6-31G*, property range is from -201.7 to 309.9 kJ, modelled in Spartan'20, Version 1.1.2.).

Table S6. Timeline of gel formation and its strength in dependance on FA concentration, and FA to Cr(III)-acetate ratio and temperature (in water:DMSO = 1:1).

FA:Cr(III)-acetate*	1:1			10:1 (0.4wt%)		20:1 (0.4wt%)		50:1 (0.4wt%)		100:1 (0.4wt%)	
Temperature	150 °C			RT	150 °C	RT	150 °C	RT	150 °C	RT	150 °C
FA content	0.1wt%	0.2wt%	0.4wt%								
Time (h) 1	A**	A	A	A	A	A	A	A	A	A	A
2	A	A	B	A	A	A	A	A	A	A	A
4	A	A	C	A	A	A	A	A	A	A	A
8	A	A	C	B	A	B	A	A	A	A	A
24	B	B	E	B	B	B	B	B	B	B	B
48	B	B	H	C	B	C	B	B	B	B	B
72	B	B	H	D	B	D	B	C	B	C	B
96	B	B	H	E	B	E	B	D	B	D	B
168	B	B	I	I	B	I	B	G	B	F	B

* Calculated to trichromium(III) cluster $[Cr_3O(O_2CCH_3)_6(OH_2)_3](O_2CCH_3)$ as one equivalent to FA (basic chromium acetate: M_W = 603.32 g/mol).
** Gel strength code by Sydansk was adapted in this case.[4]

Gel strength code by Sydansk[4]:

A No detectable gel formed: The gel appears to have the same viscosity (fluidity) as the original FA solution and no gel is visually detectable.
B Highly flowing gel: the gel appears to be only slightly more viscous (less fluid) than the initial FA solution.
C Flowing gel: most of the obviously detectable gel flows to the bottle cap upon inversion.
D Moderately flowing gel: only a small portion (about 5 to 15%) of the gel does not readily flow to the bottle cap upon inversion usually characterized as a "tonguing" gel (i.e., after hanging out of jar, gel can be made to flow back into bottle by slowly turning bottle upright).
E Barely flowing gel: the gel can barely flow to the bottle cap and/or a significant portion (>15%) of the gel does not flow upon inversion.
F Highly deformable nonflowing gel: the gel does not flow to the bottle cap upon inversion.
G Moderately deformable nonflowing gel: the gel flows about halfway down the bottle upon inversion.
H Slightly deformable nonflowing gel: the gel surface only slightly deforms upon inversion.
I Rigid gel: there is no gel-surface deformation upon inversion.
J Ringing rigid gel: a tuning-fork-like mechanical vibration can be felt after tapping the bottle (was not detected using our system).

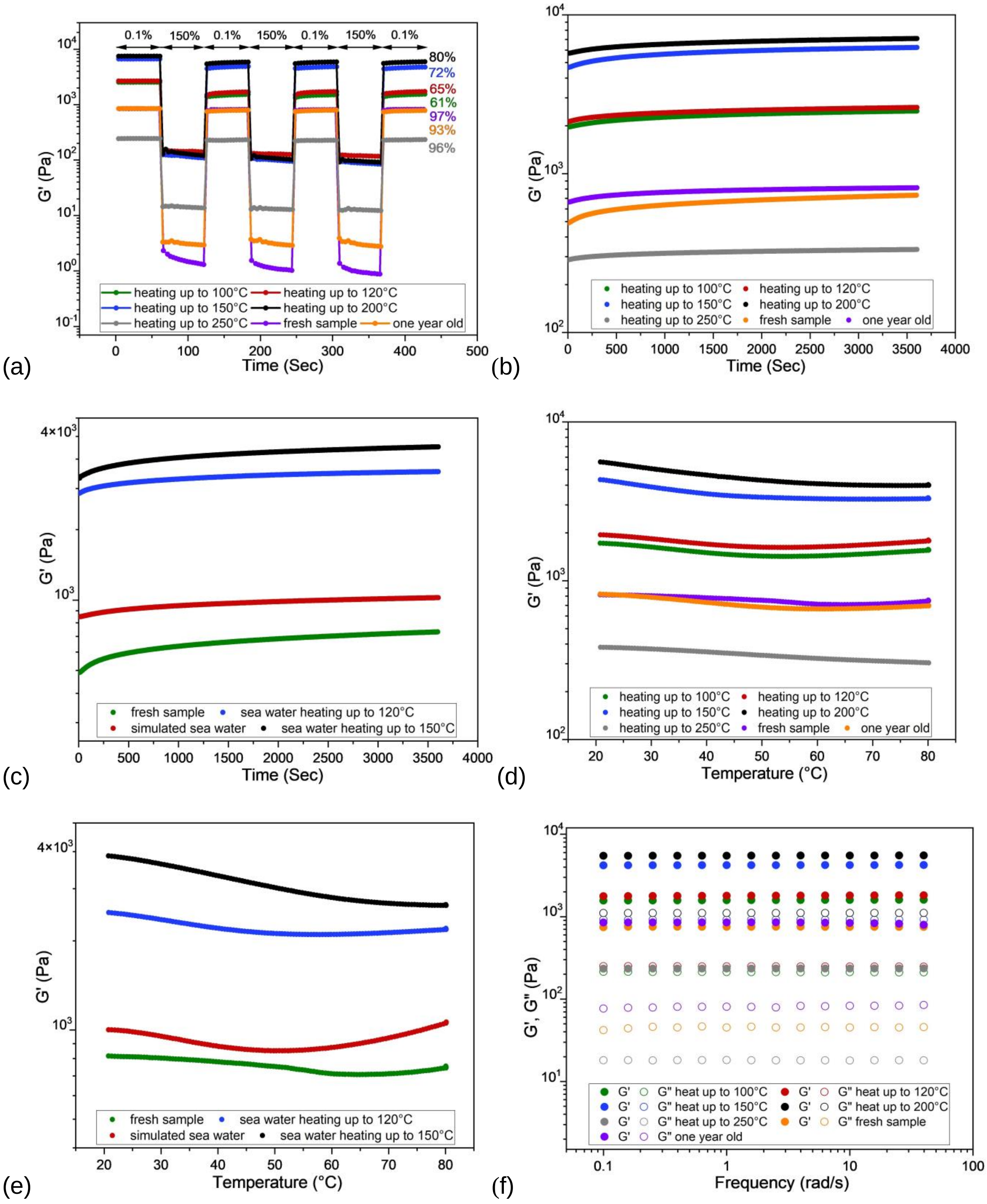
(a)
0.1% 150% 0.1% 150% 0.1% 150% 0.1%
80%
72%
65%
61%
97%
93%
96%
G' (Pa)
Time (Sec)
heating up to 100°C
heating up to 120°C
heating up to 150°C
heating up to 200°C
heating up to 250°C
fresh sample
one year old
(b)
G' (Pa)
Time (Sec)
heating up to 100°C
heating up to 120°C
heating up to 150°C
heating up to 200°C
heating up to 250°C
fresh sample
one year old
(c)
G' (Pa)
Time (Sec)
fresh sample
sea water heating up to 120°C
simulated sea water
sea water heating up to 150°C
(d)
G' (Pa)
Temperature (°C)
heating up to 100°C
heating up to 120°C
heating up to 150°C
heating up to 200°C
heating up to 250°C
fresh sample
one year old
(e)
G' (Pa)
Temperature (°C)
fresh sample
sea water heating up to 120°C
simulated sea water
sea water heating up to 150°C
(f)
G', G" (Pa)
Frequency (rad/s)
G' G" heat up to 100°C
G' G" heat up to 120°C
G' G" heat up to 150°C
G' G" heat up to 200°C
G' G" heat up to 250°C
G' G" fresh sample
G' G" one year old

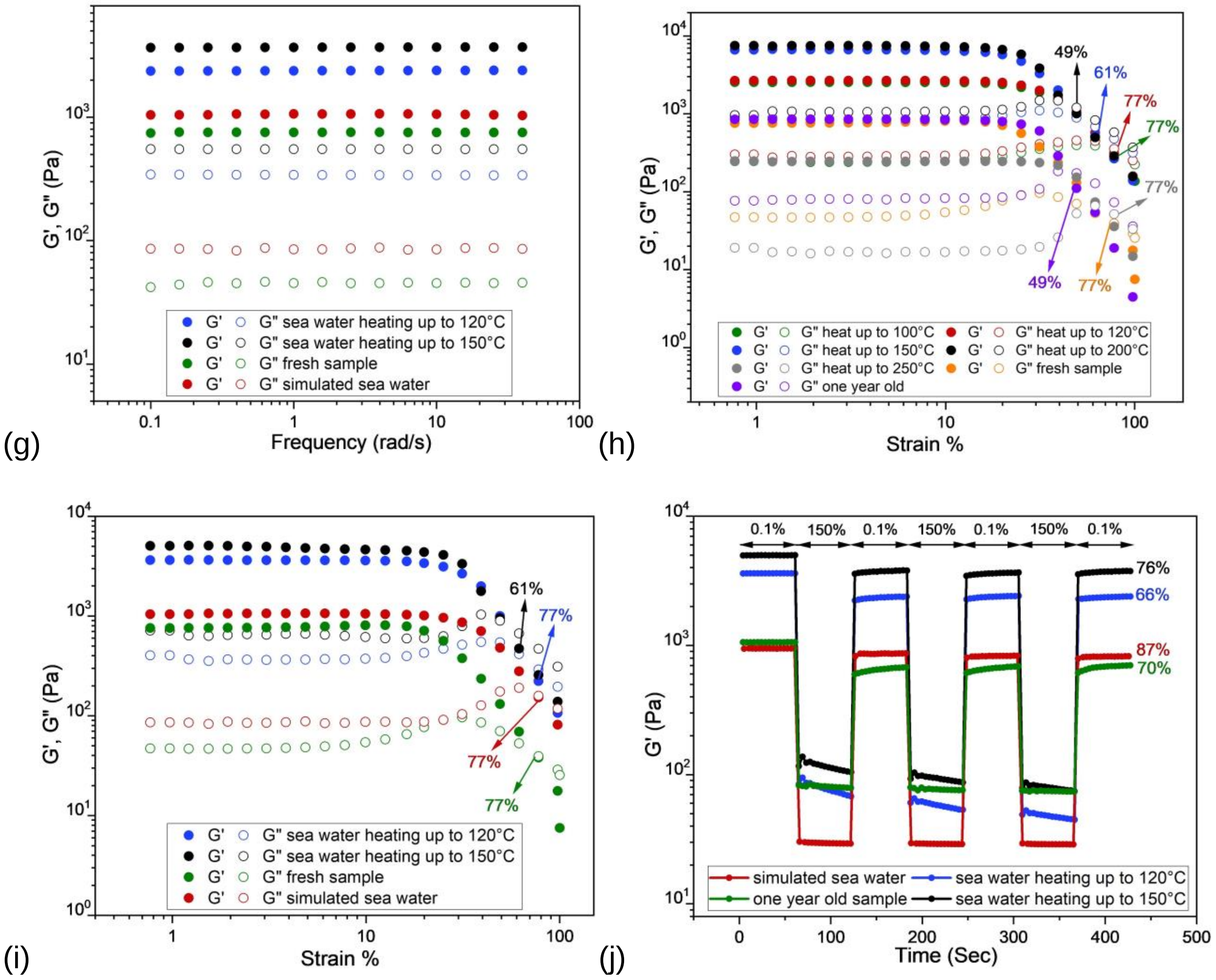

Figure S22. Rheology studies of Cr(III)-acetate metallogels (0.2 %): (a) step-strain experiment with thermally treated* gels in DMSO:H_2O solvent system; b) time sweep experiments with thermally treated gels in DMSO:H_2O solvent system; c) time sweep experiments with thermally treated gels in DMSO:seawater compared to DMSO:H_2O solvent system ("fresh sample" and "one year old sample"); d) temperature-sweep experiments with thermally treated gels in DMSO:H_2O solvent system; e) temperature-sweep experiments with thermally treated gels in DMSO:seawater compared to DMSO:H_2O solvent system; f) frequency-sweep experiments with thermally treated gels in DMSO:H_2O solvent system; g) frequency-sweep experiments with thermally treated gels in DMSO:seawater compared to DMSO:H_2O solvent system; h) strain-sweep experiments with thermally treated gels in DMSO:H_2O solvent system; i) strain-sweep experiments with thermally treated gels in DMSO:H_2O solvent system; j) step-strain experiments of thermally treated gels in DMSO:seawater compared with one year aged sample in DMSO:D_2O.
* thermally treated sample – a sample heated overnight in a closed vessel at the given temperature, cooled down, and measured at room temperature using rheometer.

Table S7. The effect of seawater and temperature on elastic modulus (G′) of FA:Cr(III)-acetate (1:1 molar ratio) gel (0.2 wt% FA in seawater/water:DMSO 1:1 by volume) measured at 20 °C.

Sample description	G′ (Pa)
Water:DMSO (fresh)	745
Water:DMSO (1 year old)	849
Seawater*:DMSO (fresh)	1044
Water:DMSO (heated at 100 °C for 12 h)	1568
Water:DMSO (heated at 120 °C for 12 h)	1774
Water:DMSO (heated at 150 °C for 12 h)	4198
Water:DMSO (heated at 200 °C for 12 h)	5478
Water:DMSO (heated at 250 °C for 12 h)	234
Seawater:DMSO (heated at 120 °C for 12 h)	2365
Seawater:DMSO (heated at 150 °C for 12 h)	3663

* A mixture of NaCl (0.48 M), $MgSO_4$ (0.03 M), $CaCl_2$ (0.01 M), and KCl (0.01 M) in distilled water (salinity 33.5 g/L).

Table S8. Timeline of FA:Cr(III)-acetate (1:1) gel formation (0.4 wt% FA in seawater:DMSO 1:1 by volume) and its strength at ambient temperature and 150 °C.

	Simulated seawater*	
Temperature	RT	150 °C
Time (hr.) 1	A*	A
2	A	A
4	B	A
8	B	A
24	C	B
48	D	B
72	E	B
96	H	C
168	I	C

* A mixture of NaCl (0.48 M), $MgSO_4$ (0.03 M), $CaCl_2$ (0.01 M), and KCl (0.01 M) in distilled water (salinity 33.5 g/L).

** Gel strength code by Sydansk was adapted in this case (see comments to Table S2).[4]

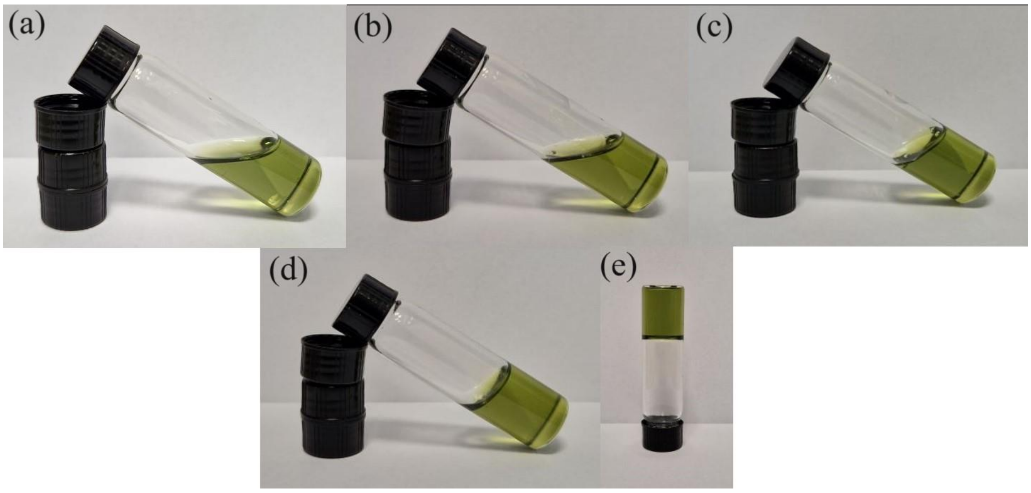

Figure S23. Time-dependence of FA-Cr(III)-acetate metallogel formation in DMSO:H_2O 1:1 system (0.2 wt%): (a) after 6 h from mixing, (b) 24 h, (c) 48 h, (d) 96 h, and (e) 120 h.

References

[1] S. Bonazzi, M. M. DeMorais, G. Gottarelli, P. Mariani, G. P. Spada, *Angew. Chem. Int. Ed.*, 1993, **32(2)**, 248-250.
[2] F. Ciuchi, G. D. Nicola, H. Franz, G. Gottarelli, P. Mariani, M. G. P. Bossi, G. P. Spada, *J. Am. Chem. Soc.*, 1994, **116(16)**, 7064-7071.
[3] G. Gottarelli, E. Mazzina, G. P. Spada, F. Carsughi, G. D. Nicola, P. Mariani, A. Sabatucci, S. Bonazzi, *Helv. Chim. Acta*, 1996, **79(1)**, 220-234.
[4] Sydansk, R. D. A New Conformance-Improvement-Treatment Chromium(lll) Gel Technology. Paper presented at the SPE Enhanced Oil Recovery Symposium, Tulsa, Oklahoma, April 1988. doi: https://doi.org/10.2118/17329-MS

Author Contributions

MAM carried out data curation, investigation, methodology, validation, visualization, writing – original draft; **SC** data curation, investigation, writing – review & editing; **SH** data curation, investigation; **N** data curation, funding acquisition, methodology, project administration, resources, supervision, visualization, writing – review & editing; **RM** funding acquisition, resources, supervision, writing – review & editing; **OJ** lead, conceptualization, data curation, funding acquisition, methodology, project administration, resources, supervision, visualization, writing – review & editing.
